# Reaction Kinetics of Green Leaf Volatiles with Sulfate, Hydroxyl, and Nitrate Radicals in Tropospheric Aqueous Phase

**DOI:** 10.1021/acs.est.1c03276

**Published:** 2021-09-28

**Authors:** Kumar Sarang, Tobias Otto, Krzysztof Rudzinski, Thomas Schaefer, Irena Grgić, Klara Nestorowicz, Hartmut Herrmann, Rafal Szmigielski

**Affiliations:** † Environmental Chemistry Group, Institute of Physical Chemistry Polish Academy of Sciences, 01-224 Warsaw, Poland; ‡ Department of Analytical Chemistry, National Institute of Chemistry, SI-1000, Ljubljana, Slovenia; § Atmospheric Chemistry Department, Leibniz Institute for Tropospheric Research, 04318, Leipzig, Germany

**Keywords:** 1-penten-3-ol, (*Z*)-2-hexen-1-ol, (*E*)-2-hexen-1-al, atmospheric radicals, atmospheric lifetime, atmospheric removal rates

## Abstract

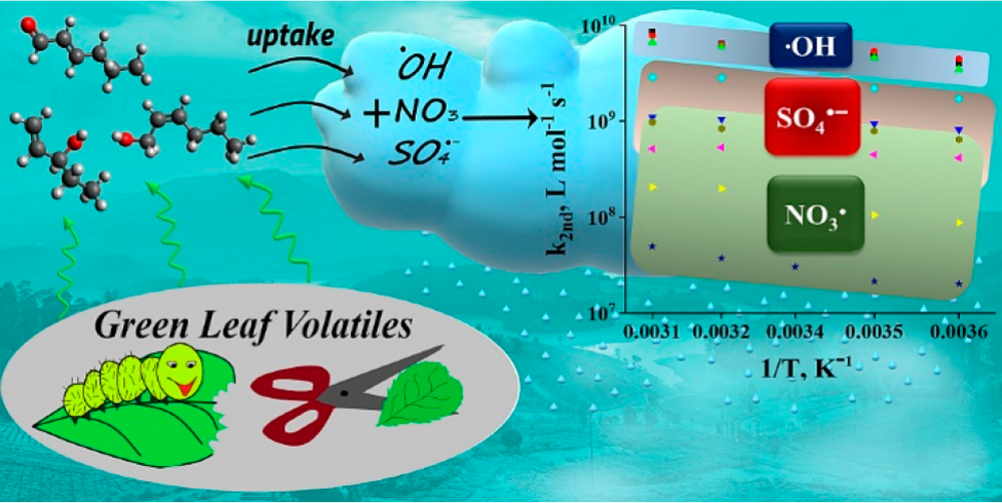

Green plants exposed to abiotic or biotic stress release C-5 and C-6 unsaturated oxygenated hydrocarbons called Green Leaf Volatiles (GLVs). GLVs partition into tropospheric waters and react to form secondary organic aerosol (SOA). We explored the kinetics of aqueous-phase reactions of 1-penten-3-ol (PENTOL), (*Z*)-2-hexen-1-ol (HEXOL), and (*E*)-2-hexen-1-al (HEXAL) with SO_4_^•–^, ^•^OH, and NO_3_^•^. At 298 K, the rate constants for reactions of PENTOL, HEXOL, and HEXAL with SO_4_^•–^ were, respectively, (9.4 ± 1.0) × 10^8^ L mol^–1^ s^–1^, (2.5 ± 0.3) × 10^9^ L mol^–1^ s^–1^, and (4.8 ± 0.2) × 10^8^ L mol^–1^ s^–1^; with ^•^OH – (6.3 ± 0.1) × 10^9^ L mol^–1^ s^–1^, (6.7 ± 0.3) × 10^9^ L mol^–1^ s^–1^, and (4.8 ± 0.3) × 10^9^ L mol^–1^ s^–1^; and with NO_3_^•^ – (1.5 ± 0.15) × 10^8^ L mol^–1^ s^–1^, (8.4 ± 2.3) × 10^8^ L mol^–1^ s^–1^, and (3.0 ± 0.7) × 10^7^ L mol^–1^ s^–1^. The rate constants increased weakly with temperatures ranging from 278 to 318 K. The diffusional limitations of the rate constants appeared significant only for the GLV–^•^OH reactions. The aqueous-phase reactions appeared negligible in deliquescent aerosol and haze water but not in clouds and rains. The atmospheric lifetimes of GLVs decreased from many days to hours with increasing liquid water content and radicals’ concentration.

## Abstract 2

Green leaf volatiles emitted by plants under biotic and abiotic stress react with atmospheric oxidants in the gas and water phases to influence air quality.

## Introduction

The impact of Volatile Organic Compounds (VOCs) on the air quality and formation of ozone in the troposphere became recognized in the 1950s.^1^ Their crucial role as precursors of Secondary Organic Aerosol (SOA) was noticed in 1960^2^ and earned global attention in the 1990s.^3^ The contribution of SOA to the Earth’s solar radiative budget, climate change, and cloud formation, as well as its impact on human health through gas- and aqueous-phase processes, have been progressively investigated.^4−6^ Still, however, the vast fraction of potentially important SOA sources and transformation processes remain unknown.^5,7,8^ Hydrophilic aerosol particles serve as Cloud Condensation Nuclei (CCN) and promote cloud formation playing a significant role in cooling the Earth’s climate.^4^ Among biogenic VOCs (BVOCs), isoprene^9−15^ and monoterpenes^16−20^ have already gained ample attention, while green leaf volatiles (GLVs), also potentially precursors of SOA,^21^ have been much less studied.

GLVs are unsaturated C-5 and C-6 compounds produced from fatty acids present in plant leaves, e.g., α-linolenic and linoleic acids.^22,23^ GLVs are released into the atmosphere when plants experience stresses of varying nature such as cell damage or wounding.^24,25^ A considerable amount of data exists on the oxidation of GLVs in the gas phase.^26−35^ Still, very few studies have considered their reactions in the aqueous phase leading to the formation of SOA.^36−38^ Rain, cloud droplets, fog, and aerosol liquid water (ALW) over the vegetation can take up GLVs, promoting their aqueous-phase oxidation to less volatile compounds. The droplets eventually dry out, leaving behind the SOA particles. Richards-Henderson et al.^37^ studied the oxidation of five GLVs ((*Z*)-3-hexen-1-ol, (*Z*)-3-hexenyl acetate, methyl salicylate, methyl jasmonate, and 2-methyl-3-butene-2-ol) by ^•^OH radicals in aqueous solutions. The obtained second-order rate constants (*k*_second_) were nearly diffusion-limited (∼10^9^ L mol^–1^ s^–1^) and weakly dependent on temperature with average activation energy (*E*_a_) lower than 15 kJ mol^–1^. The reactions of methyl jasmonate, methyl salicylate, (*Z*)-3-hexenyl acetate, (*Z*)-3-hexen-1-ol, and 2-methyl-3-butene-2-ol with organic triplet excited states and with singlet oxygen were significantly slower at 298 K^39^ (*k*_second_ = (0.13–22) × 10^8^ L mol^–1^ s^–1^ and (8.2–60) × 10^5^ L mol^–1^ s^–1^, respectively). Hansel et al.^38,40^ identified several low-volatile products of the aqueous-phase oxidation of methyl jasmonate and methyl salicylate by ^•^OH and proposed formation mechanisms. Shalmazari et al.^30^ identified organosulfates in SOA produced from the atmospheric oxidation of 2-*E*-pentenal, 2-*E*-hexenal, and 3-*Z*-hexenal. Barbosa et al.^41^ studied the oxidation of (*Z*)-3-hexen-1-ol oxidation by ^•^OH and O_3_. Liyana-Arachchi et al.^42,43^ studied theoretically and experimentally the adsorption of methyl salicylate, 2-methyl-3-buten-2-ol, (*Z*)-3-hexen-1-ol, and (*Z*)-3-hexenyl acetate on air–water interfaces.

**Scheme 1:**
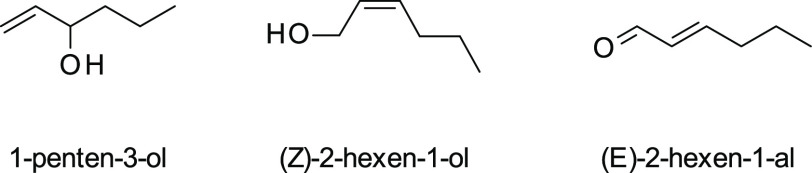
GLVs Studied in This Work

Researching novel atmospheric compounds requires understanding their kinetics to predict their fate in the atmosphere.^44,45^ In this work, we explored for the first time the kinetics of aqueous-phase reactions of three GLVs—1-penten-3-ol (PENTOL), (*Z*)-2-hexen-1-ol (HEXOL), and (*E*)-2-hexen-1-al (HEXAL) (Scheme 1)—with tropospheric radicals ^•^OH, SO_4_^•–^, and NO_3_^•^. Our main goal was to determine the rate constants and evaluate the atmospheric significance of the reactions. The examined GLVs may be effective precursors of aqueous SOA formation like other GLV,^37^ even though they are moderately water-soluble and intermediary volatile. Their physical properties were estimated using the EPI suite 2012 from EPA^46^ (Table S1). The global annual emission of GLVs (hexenal, hexenol, and hexenyl acetate) is 10–50 Tg C/yr,^47^ giving rise to 1–5 Tg C/yr SOA, i.e., at least one-third of that from isoprene.^48^ The local concentrations of the several GLVs, including 1-penten-3-ol, in the vicinity of stressed plants reach a few ppb.^49,50^ Heiden et al.^51^ and Jardine et al.^25^ observed the high emission of many GLVs, including 1-penten-3-ol and (*Z*)-2-hexenal, under stress from pathogen attack or ozone exposure. Common anthropogenic activities like harvesting the cereal and biofuel grasses or residential grass mowing cause significant GLVs emissions that influence the local air quality.^52−54^ Novel agricultural, horticultural, and forestry practices based on the fumigation of plants with GLVs for better resistance against pathogens and abiotic stress will probably increase the GLV emissions.^55,56^ Thus, GLVs chemistry can play an essential role in the atmosphere and requires thorough attention in atmospheric chemistry and air quality models. Our work increases the chemical-kinetic database for the aqueous-phase reactions that is indispensable for such modeling, as generally postulated in several major reviews.^4,57,58^

## Experimental Methods

### Chemicals

All chemicals were purchased and used without further purification: 1-penten-3-ol (Sigma-Aldrich, 99.0%), (*Z*)-2-hexen-1-ol (Sigma-Aldrich, 95.0%), (*E*)-2-hexen-1-al (Sigma-Aldrich, 98.0%), sodium persulfate (Na_2_S_2_O_8_, Sigma-Aldrich and Honeywell, 99.0%), sodium nitrate (NaNO_3_, EMSURE, 99.5%), hydrogen peroxide (H_2_O_2_, CHEMSOLUTE, 30.0% wt. in H_2_O), potassium thiocyanate (KSCN, CHEMSOLUTE, 99.0%). Aqueous solutions were freshly prepared using Milli-Q water (18.2 MΩ cm, TOC < 5 ppb).

### Kinetic Experiments

The Laser Flash Photolysis-Laser Long Path Absorption (LFP-LLPA, Figure S1) was used to measure the rates of the aqueous-phase oxidation of GLVs by the relevant radicals. A detailed description of the setup is available elsewhere.^57,59^

The LFP-LLPA method applied is like that used by Schöne et al. and Otto et al.^60,61^ A freshly prepared aqueous solution containing a GLV compound and radical precursors was transferred into the solution tank. The solution flowed down through the measurement cell (4 cm × 3.5 cm × 2 cm) thermostated with Water Thermostat (Julabo or S LAUDA). In the measurement cell, the radical precursors’ photolysis occurred by excimer laser (COMPEX 201 series) pulses of microsecond width triggered at 4 Hz (DG535 Digital Delay Generator, Standford Research Systems). A continuous-wave (CW) laser measured the radicals’ light absorption after passing the beam across the cell several times by a White mirror setup. The signal’s final intensity was measured with a photodiode and recorded with an oscilloscope (Data SYS 944, Gould) and a computer for further data processing to obtain a second-order rate constant *k*_second_ of the reaction. The temperature of measurements was constant and varied from 278 to 318 K. The GLVs studied do not undergo ion speciation, so all experiments were carried out at pH close to 7.

Table S4 shows the LFP-LLPA configuration, while Table S5 shows the initial concentrations of GLVs and radical precursors. Figure S2 and Table S2 present the recorded UV spectra and calculated molar absorption coefficients of the GLVs. For experiments with PENTOL and HEXOL, the 248 nm excimer laser and 407 nm CW laser (LSR 407 nm, Coherent) were used to generate the ^•^OH and SO_4_^•–^ radicals and follow the reactant concentrations, respectively. Due to the strong absorption of light by HEXAL at 248 nm (ε_248 nm_ = 1722.1 L mol^–1^ cm^–1^), a 308 nm excimer laser and 473 nm CW laser (LasNova Series 40 blue, LASOS) were used for exploring the kinetics of HEXAL reactions with ^•^OH and SO_4_^•–^ (ε_308 nm_(HEXAL) = 51.8 L mol^–1^ cm^–1^). The 473 nm CW laser secured better light absorption at low concentrations of radicals. Figure S3 shows a typical absorbance time trace in the experiments following a laser flash photolysis. The NO_3_^•^ kinetics for all the three GLVs was followed using 351 nm excimer laser and 635 nm continuous-wave laser (Radius, Coherent).

### SO_4_^•–^ Kinetics

For exploring the reactions of SO_4_^•–^ radical-anions with GLVs, an aqueous solution of Na_2_S_2_O_8_ and a GLV was photolyzed using an excimer laser (248 nm for PENTOL, HEXOL, 308 nm for HEXAL) to generate SO_4_^•–^ by dissociating the S_2_O_8_^2–^ ions. Each intensity vs time plot (Figure S3) was the average of eight separate recordings. The intensity was converted to the concentration of SO_4_^•–^ radicals using the molar extinction coefficients (ε_407 nm_ = 1260 L mol^–1^ cm^–1^ and ε_473 nm_ = 1389 L mol^–1^ cm^–1^).^62^ A pseudo-first-order rate constant *k*_first_ for the reaction was calculated from the slope of the concentration vs time plot. The pseudo-first-order *k*_first_ constants were plotted against the initial concentrations of the GLV to obtain the second-order rate constant *k*_second_ for the reaction.^60,61^

### ^•^OH Kinetics

Because of weak light absorption and spectra of ^•^OH overlapping with those of the organic constituents, ^•^OH radicals are difficult to detect directly.^62−64^ Therefore, the competition kinetics method^65^ was employed to explore the kinetics of ^•^OH radical reactions with GLVs. H_2_O_2_ was photolyzed at 248 nm (PENTOL, HEXOL) or 308 nm (HEXAL) to produce ^•^OH with KSCN added as a reference compound. The reactions a–e occurred, where reaction e is the sink of the SCN radical anion:
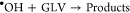
a
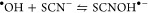
b
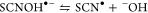
c
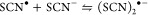
d
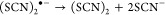
eThe dithiocyanate radical-anion ((SCN)_2_^•–^) strongly absorbs light in the visible region of the spectrum (400–550 nm).^62^ The solution’s absorbance was measured using a CW laser at 407 nm for PENTOL and HEXOL and at 473 nm for HEXAL. The *k*_second_ for the reaction GLV + ^•^OH was calculated using eq 1 from Schaefer and Herrmann^66^ and eq 2 from Zhu et al.^67^
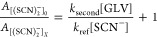
1
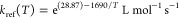
2where *A*_[(SCN)_2_^–^]_0__ is the absorbance of (SCN)_2_^•–^ in the absence of GLV, *A*_[(SCN)_2_^–^]_*X*__ is the absorbance of (SCN)_2_^•–^ in the presence of GLV at the concentration *X* in the reaction solution, *k*_ref_ is the reference rate constant for reaction b. The GLV compound absorbs UV light at both wavelengths of excimer laser (248 nm for PENTOL and HEXOL, and 308 nm for HEXAL). Thus, GLV act as internal filters of the UV light and reduce the initial ^•^OH concentrations measured in experiments. Therefore, *A*_[(SCN)_2_^–^]_0__ in eq 1 was corrected for each GLV at all temperatures using the procedure of Schaefer and Herrmann.^66^Table S3 shows the changes in the initial ^•^OH concentrations (<0.05% for PENTOL, 0.2–0.9% for HEXOL, and 1–3% for HEXAL).

### NO_3_^•^ Kinetics

The reaction of GLV with NO_3_^•^ radicals started by the photolysis of solutions containing NaNO_3_, Na_2_S_2_O_8_, and a GLV in the measurement cell, using 351 nm excimer laser. The NO_3_^•^ radicals were generated by reactions f and g.^59,60,68^ Light absorbance by NO_3_^•^ was measured using a red diode CW laser (635 nm) and converted to concentrations using the molar extinction coefficient ε_635 nm_ = 1120 M^–1^ cm^–1^.^69^ The intensity–time traces were processed using the same method as for SO_4_^•–^ kinetics to get the *k*_second_ (GLV + NO_3_^•^).
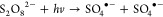
f
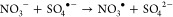
gThe uncertaintiy of each *k*_second_ determined in the present study was calculated as a product of the standard deviation and the Student’s *t*-factor taken with the 95% confidence level. Each rate constant determined for a single GLV at a single temperature was backed by 40 measurements (8 replicates for a single GLV concentration).

### Diffusion Limitations of Rate Constants

The radical reactions with *k*_second_ on the order of 10^9^ L mol^–1^ s^–1^ or higher can be controlled by the diffusion of reactants, at least in part. Therefore, we analyzed the experimental rate constants (i.e., the constants obtained from the LFP-LLPA experiments, *k*_obs_) for diffusion limitations using a simple resistance-in-series approach^70^ to split them into the true rate constants (*k*_reac_) and the rate constants for the diffusion of reactants (*k*_diff_):
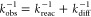
3
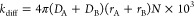
4where all *k* are the second-order rate constants (L mol^–1^ s^–1^), *D* is a diffusion coefficient of reactants A and B (m^2^ s^–1^), *r* is the radius of reactant molecules A and B (m), and *N* is the Avogadro number (for details, see Section S6, SI).

### Kinetic Modeling of Reactions

We used the COmplex PAthway SImulator of biochemical systems (COPASI from Bioinformatics,^71^ to evaluate the bias of the rate constants determined for reactions of GLV with nitrate radicals. We chose the evolutionary programming method (number of generations 200, population size 20) for parameter estimation and the deterministic ordinary differential equation solver (LSODA)^71−73^ for simulating the reaction time courses.

## Results and Discussion

### Reactions of SO_4_^•–^ Radical-Anions with PENTOL, HEXOL, and HEXAL

**Figure 1:**
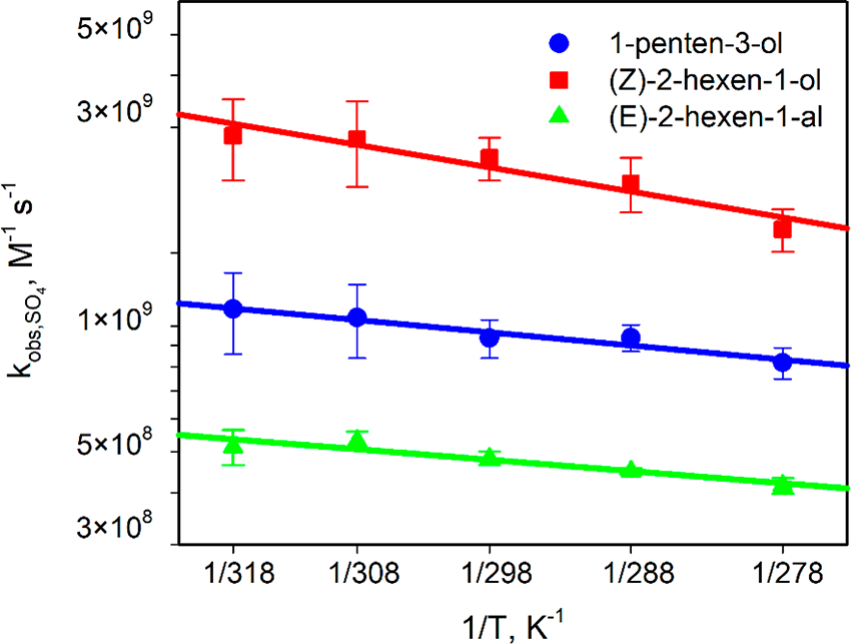
Experimental rate constants for reactions of GLVs with SO_4_^•–^ at various temperatures (coefficient of determination *R*^2^ = 0.94 for 1-penten-3-ol, 0.92 for (*Z*)-2-hexen-1-ol, and 0.90 for (*E*)-2-hexen-1-al; Table S7, eqs 5–7).

Previous studies^74^ showed that SO_4_^•–^ radical is a strong oxidizing agent and reacts with many organic compounds at the rates nearly controlled by the diffusion of reactants. The experimental rate constants (*k*_obs_) for the aqueous-phase reactions of PENTOL, HEXOL, and HEXAL with SO_4_^•–^ determined in this study at 278–318 K range from (4.2 ± 0.2) × 10^8^ to (2.9 ± 0.6) × 10^9^ L mol^–1^ s^–1^ (Table S7). The Arrhenius plots (Figure 1) and eqs 5–7 show the rate constants weakly increase with temperature.
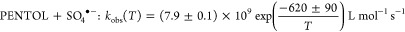
5
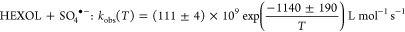
6
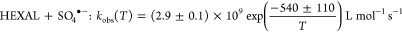
7The second-order rate constants corrected for the diffusional limitations (*k*_reac_) are only slightly higher and range from (4.4 ± 0.2) × 10^8^ to (3.7 ± 0.8) × 10^9^ L mol^–1^ s^–1^ (Table S7, Figure S4a). The contribution of diffusion to the experimental rate constant (% *k*_diff_) is about 9% for PENTOL, 19–23% for HEXOL, and 4–5% for HEXAL (Table S7). Thus, the reactions of GLVs with the SO_4_^•–^ are mostly chemically controlled.

### Reactions of ^•^OH Radicals with PENTOL, HEXOL, and HEXAL

**Figure 2:**
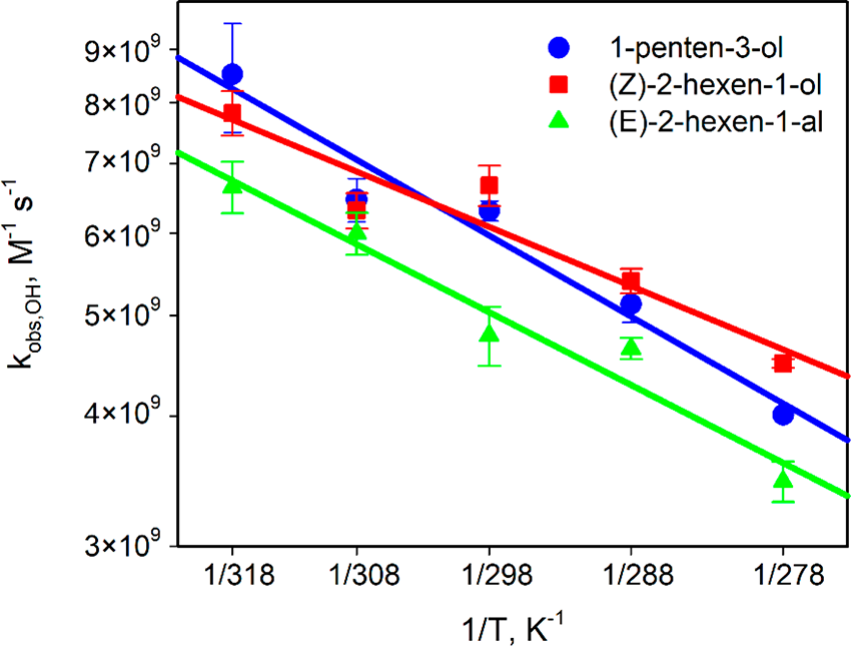
Experimental rate constants for reactions of GLVs with ^•^OH at various temperatures (coefficient of determination *R*^2^ = 0.96 for 1-penten-3-ol, 0.90 for (*Z*)-2-hexen-1-ol, and 0.96 for (*E*)-2-hexen-1-al; Table S8, eqs 8–10).

Figures 2 and S4b show the temperature dependence of the experimental (*k*_obs_) and diffusion-corrected (*k*_reac_) rate constants for the aqueous-phase reactions of GLVs with ^•^OH. The experimental constants weakly increase with temperature from (3.5 ± 0.2) × 10^9^ to (8.5 ± 1.0) × 10^9^ L mol^–1^ s^–1^ (Figure 2, eqs 8–10, Table S8).
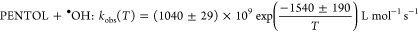
8
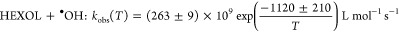
9
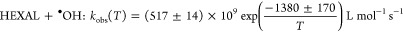
10The constants are larger than for reactions with SO_4_^•–^ and closer to the diffusional limit ((4.9 ± 0.2) × 10^9^ to (16.4 ± 0.8) × 10^9^ L mol^–1^ s^–1^, Table S8). The diffusion contribution to *k*_obs_ is about 35–57% for PENTOL, 39–52% for HEXOL, and 30–45% for HEXAL (Table S8). Thus, the diffusion of reactants significantly influenced the experimental reaction rates.

### Reactions of NO_3_^•^ Radicals with PENTOL, HEXOL, and HEXAL

**Figure 3:**
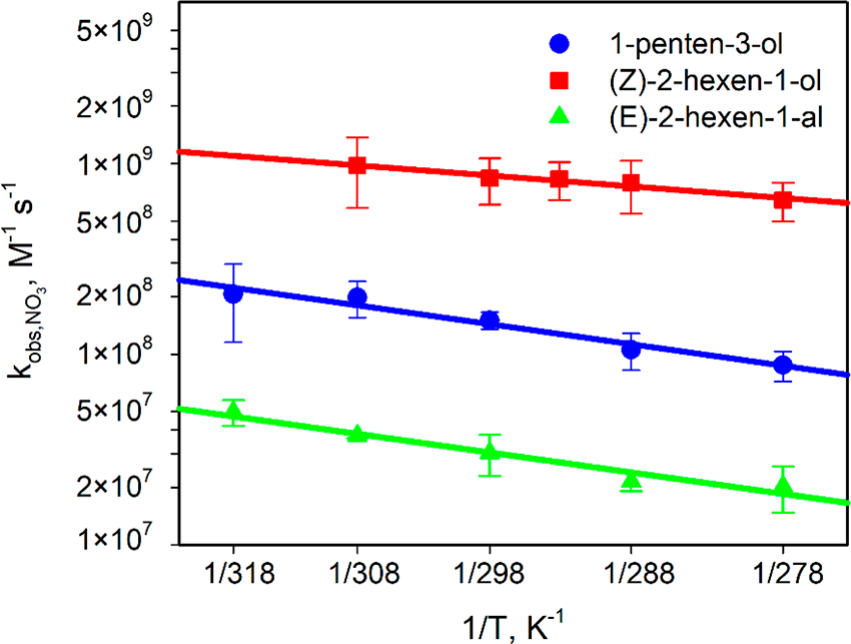
Experimental rate constants for reactions of GLVs with NO_3_^•^ at various temperatures (coefficient of determination *R*^2^ = 0.93 for 1-penten-3-ol, 0.94 for (*Z*)-2-hexen-1-ol, and 0.96 for (*E*)-2-hexen-1-al; Table S9, eqs 11–13).

The rate constants for the aqueous-phase reactions of PENTOL, HEXOL, and HEXAL with NO_3_^•^ radicals range from (2.0 ± 0.6) × 10^7^ to (9.8 ± 3.9) × 10^8^ L mol^–1^ s^–1^ (Figure 3, Table S9). We could not determine the rate constant for HEXOL at 318 K, so we got the fifth value at 293 K. Figure 3 and eqs 11–13 show the temperature variation of the experimental rate constants.
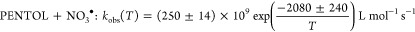
11
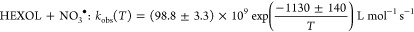
12
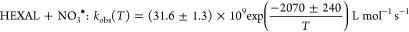
13The contribution of diffusion to *k*_obs_ was 1–2% for PENTOL, 7–8% for HEXOL, and 0.2–0.4% for HEXAL (Table S9, Figure S4c), so all those reactions are fully chemically controlled. Large error bars in the Arrhenius plots result from low light absorption values measured in the experiments but still fall within the 95% confidence interval.

The magnitude of the rate constants for the aqueous-phase reactions of GLV with radicals was 10^9^ for ^•^OH, 10^8^ for SO_4_^•–^, and 10^7^ L mol^–1^ s^–1^ for NO_3_^•^. Only for HEXOL, the rate constant for NO_3_^•^ was larger than for SO_4_^•–^. HEXOL appeared the fastest reacting compound of the three GLV studied. The possible explanation is that the HEXOL molecule has a C=C double bond position available for radical addition and two allylic positions available for H-abstraction by the radicals, while PENTOL and HEXAL have one C=C position and only one allylic position available. The difference in the rate constants between HEXOL and PENTOL or HEXAL is more prominent in the case of SO_4_^•–^ and NO_3_^•^ reactions, as they are more chemically controlled than the partially diffusion-controlled reactions with ^•^OH.

Table 1 shows the rate constants and activation energies for aqueous-phase reactions of several GLVs and structurally similar compounds with ^•^OH.^37,57,62^ The activation energies range from 6 to 17 kJ mol^–1^, and the rate constants from 2 × 10^9^ to 8.3 × 10^9^ L mol^–1^ s^–1^. The rate constants for 1-penten-3-ol, (*Z*)-2-hexen-1-ol, and (*E*)-2-hexen-1-al with ^•^OH determined in this study at 298 K are relatively close to the rate constants of other GLV ((*Z*)-3-hexen-1-ol, (*Z*)-3-hexenyl acetate, 2-methyl-3-buten-2-ol, and methyl jasmonate) reported by Richards-Henderson et al. (Table 1).^37^

**Table 1:** Rate Constants and Activation Energies for Reactions of GLVs and Structurally Similar Organic Compounds with ^•^OH

	*k*_298 K_	*E*_A_		
compound	10^9^ L mol^–1^ s^–1^	kJ mol^–1^	method	ref
(*Z*)-3-hexen-1-ol	5.3 ± 0.2	12 ± 0.3	a	(37)
(*Z*)-3-hexenyl acetate	8.3 ± 0.6	17 ± 2	a	(37)
2-methyl-3-butene-2-ol	7.3 ± 0.7	13 ± 2	a	(37)
methyl jasmonate	6.8 ± 0.5	15 ± 2	a	(37)
methyl salicylate	8.1 ± 0.6	14 ± 1	a	(37)
methyl isobutyl ketone	2.0 ± 0.2	10 ± 2	b	(75)
isobutyraldehyde	2.9 ± 1.0	6 ± 3	b	(76)
1-penten-3-ol	6.3 ± 0.1	13 ± 2	c	This work
(*Z*)-2-hexen-1-ol	6.7 ± 0.3	9 ± 2	c	This work
(*E*)-2-hexen-1-al	4.8 ± 0.3	12 ± 1	c	This work

a Competition kinetics.

b Static photo reactor/Fenton for OH.

c LFP-LLPA/photolysis of H_2_O_2_ (competition kinetic, reference compound SCN^–^)

The rate constants and activation energies for the reactions of structurally similar oxygenated compounds with ^•^OH radicals, such as methyl isobutyl ketone and isobutyraldehyde, are also similar to those for GLV (Table 1).

No aqueous-phase kinetic data existed by now for the reactions of GLVs with SO_4_^•–^ and NO_3_^•^. However, data for structurally similar compounds were reviewed by Herrmann et al., and Neta and Huie.^57,62,74^ For instance, Schöne et al. investigated the temperature-dependent kinetics of methacrolein (MAC) and methyl vinyl ketone (MVK).^77^ The rate constants for MAC and MVK at 298 K were as follows: (9.4 ± 0.7) × 10^9^ L mol^–1^ s^–1^ and (7.3 ± 0.5) × 10^9^ L mol^–1^ s^–1^ for ^•^OH; (9.9 ± 4.9) × 10^7^ L mol^–1^ s^–1^ and (1.0 ± 0.2) × 10^8^ L mol^–1^ s^–1^ for SO_4_^•–^; (4.0 ± 1.0) × 10^7^ L mol^–1^ s^–1^ and (9.7 ± 3.4) × 10^6^ L mol^–1^ s^–1^ for NO_3_^•^, respectively. Those rate constants were similar by order of magnitude to the rate constants for three GLVs determined in this study.

### Bias of the Experimental Rate Constants for Reactions of GLV with NO_3_^•^

The experimental method used to determine the rate constants assumed that NO_3_^•^ radicals were consumed only in the reaction with a GLV. However, NO_3_^•^ can participate in other reactions, e.g., with ^–^OH, H_2_O, HO_2_^•^, S_2_O_8_^2–^, and organic peroxides that form by the autoxidation of alkyl compounds produced by the reaction of GLV with SO_4_^•–^ radicals. To assess the influence of “neglected” reactions, we constructed a chemical-kinetic Model_1 including those reactions and used it to evaluate the rate constants for GLV+ NO_3_^•^ reactions (for details, see Section 8, SI). Table 2 compares the experimental rate constants, experimental uncertainties from the LFP-LLPA procedure, and model-derived rate constants. The relative algebraic difference between the constants (eq 14) estimates the bias of the experimental constants due to the “neglected” sinks of NO_3_^•^
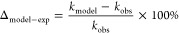
14The bias is the largest for HEXAL, which reacts with NO_3_^•^ most slowly among the GLVs studied. The experimental rate constants are overestimated by 6–25%. The effect decreases with temperature, probably due to the relative acceleration of the HEXAL – NO_3_^•^ reaction. For PENTOL and HEXOL, the bias is smaller, with the rate constants overestimated by less than 15%. In most cases, the intrinsic uncertainty of the experimental rate constants determined by the LFP-LLPA procedure is significantly larger than the bias due to the “neglected” NO_3_^•^ sinks, including the reactions with peroxy intermediates. Few exceptions occurred for the slowest reacting HEXAL at 288 and 308 K. Probably, the data-processing unit of the LFP-LLPA method can be modified based on the present results to reduce the bias for the rate constants of reactions with NO_3_ radicals.

**Table 2:** Experimental and Model-Derived Rate Constants (L mol^–1^ s^–1^) for Reactions between GLV and NO_3_^•^

	278 K	288 K	298 K	308 K	318 K
PENTOL					
Model_1	9.3 × 10^7^	9.3 × 10^7^	1.4 × 10^8^	1.7 × 10^8^	
Experimental (*k*_obs_)	8.8 × 10^7^	1.1 × 10^8^	1.5 × 10^8^	2.0 × 10^8^	
Δ_Model-Exp_, %	+5	–13	–7	–14	
Experimental uncertainty, %	±18	±22	±10	±21	
HEXOL					
Model_1	6.6 × 10^8^	7.8 × 10^8^	7.7 × 10^8^	8.5 × 10^8^	
Experimental (*k*_obs_)	6.4 × 10^8^	7.9 × 10^8^	8.4 × 10^8^	9.8 × 10^8^	
Δ_Model-Exp_, %	+3	–1	–9	–15	
Experimental uncertainty, %	±23	±31	±23	±27	
HEXAL					
Model_1	1.6 × 10^7^	1.7 × 10^7^	2.6 × 10^7^	3.5 × 10^7^	4.5 × 10^7^
Experimental (*k*_obs_)	2.0 × 10^7^	2.1 × 10^7^	3.0 × 10^7^	3.7 × 10^7^	5.0 × 10^7^
Δ_Model-Exp_, %	–25	–24	–15	–6	–10
Experimental uncertainty, %	±27	±11	±24	±3	±15

### Activation Parameters

The activation parameters are essential in understanding the chemical mechanisms of reactions. Table 3 presents the activation parameters for reactions of GLVs with atmospheric radicals (SO_4_^•–^, ^•^OH, and NO_3_^•^). The equations for activation enthalpies (Δ*H*^‡^), activation entropies (Δ*S*^‡^), Gibb’s activation energy (Δ*G*^‡^) are shown in the SI, while the calculation procedure is described elsewhere.^60,61,78^ All Arrhenius plots obtained are linear (Figures 1–3) and follow eq 15
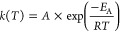
15where *k* is the rate constant, *E*_A_ is the activation energy, *A* is the pre-exponential factor, *R* is the gas constant, and *T* is the absolute temperature. The Arrhenius expressions for the aqueous-phase reactions of PENTOL, HEXOL, and HEXAL with SO_4_^•–^, ^•^OH, and NO_3_^•^ are provided for the first time (eqs 5–13). The ratio of *E*_A_ to the average kinetic energy (*RT*) directly influences the reaction rate constant. The *E*_A_ values lower than 18 kJ mol^–1^ explain the weak temperature dependence of the reactions rates. The low activation enthalpies Δ*H*^‡^ (2 to 15 kJ mol^–1^) and negative activation entropies Δ*S*^‡^ (−72 to −23 J mol^–1^ K^–1^) explain the decreasing randomness of molecules within the system. They indicate that the aqueous-phase reactions studied mainly proceed via the radical addition to the double bond or associative pathway and warrant further theoretical and experimental investigation. The activation parameters for the diffusion-corrected rate constants were calculated following a similar procedure, and the activation energies are still less than 20 kJ mol^–1^ (Table S10).

**Table 3:** Experimentally Determined Activation Parameters for the Reactions of GLVs with SO_4_^•–^, ^•^OH, and NO_3_^•^ Radicals

		*E*_A_	*A*	Δ*H*^‡^	*–*Δ*S*^‡^	Δ*G*^‡^
reactants		kJ mol^–1^	L mol^–1^ s^–1^	kJ mol^–1^	J mol^–1^ K^–1^	kJ mol^–1^
SO_4_^•–^	PENTOL	5 ± 1	(7.9 ± 0.1) × 10^9^	3 ± 1	64 ± 1	22 ± 4
	HEXOL	10 ± 2	(1.1 ± 0.1) × 10^11^	7 + 12/–7	42 ± 1	20 ± 5
	HEXAL	4 ± 1	(2.9 ± 0.1) × 10^9^	2 ± 1	72 ± 2	24 ± 6
^•^OH	PENTOL	13 ± 2	(10.4 ± 0.3) × 10^11^	10 ± 2	23 ± 1	17 ± 3
	HEXOL	9 ± 2	(2.6 ± 0.1) × 10^11^	7 ± 2	35 ± 1	17 ± 4
	HEXAL	12 ± 1	(5.2 ± 0.1) × 10^11^	9 ± 1	29 ± 1	18 ± 3
NO_3_^•^	PENTOL	17 ± 2	(1.5 ± 0.1) × 10^11^	15 ± 2	39 ± 2	27 ± 5
	HEXOL	9 ± 1	(3.8 ± 0.1) × 10^10^	7 ± 1	51 ± 1	22 ± 4
	HEXAL	17 ± 2	(3.1 ± 0.1) × 10^10^	15 ± 2	52 ± 2	30 ± 6

## Atmospheric Implications

Estimating GLV fluxes removed from the atmosphere by gas-phase reactions, aqueous-phase reactions, and other processes like deposition to land and aquatic ecosystems requires extensive modeling of individual scenarios beyond the scope and size of this paper. To estimate the proportion of the gas-phase and aqueous-phase fluxes, we scaled the GLV removal rates dividing them by the concentration of GLV. That descriptor compares the corresponding GLV fluxes independent of the GLV concentration. Besides, we evaluated the atmospheric significance of gas-phase and aqueous-phase reactions of GLV with radicals using the commonly accepted method of atmospheric lifetimes and relative rates of removal.

### Scaled GLV Removal Rates

Table 4 shows the scaled removal rates due to gas-phase and aqueous-phase reactions of PENTOL, HEXOL, and HEXAL with ^•^OH, NO_3_^•^, and SO_4_^•–^ in dry air, urban clouds, remote clouds, and urban aerosol (SO_4_^–^ radicals occur only in the aqueous phase). The calculation was based on equations S12–S13 and data in Tables S7–S9, S11, and S12.

**Table 4:** Scaled Removal Rates of GLV from the Atmosphere Due to Gas-Phase and Aqueous-Phase Reactions with ^•^OH, NO_3_^•^, and SO_4_^•–^ at 298 K

		ω	scaled removal rates, s^–1^		
system	sink	m^3^ m^–3^	PENTOL	HEXOL	HEXAL
Urban clouds	Gas-phase reactions	0	3 × 10^–7^	3 × 10^–6^	2 × 10^–6^
	Aqueous-phase reactions	1 × 10^–8^	1 × 10^–9^	6 × 10^–9^	6 × 10^–11^
		1 × 10^–6^	1 × 10^–7^	6 × 10^–7^	6 × 10^–9^
Remote clouds	Gas-phase reactions	0	1 × 10^–6^	1 × 10^–5^	1 × 10^–5^
	Aqueous-phase reactions	1 × 10^–8^	4 × 10^–9^	7 × 10^–9^	3 × 10^–10^
		1 × 10^–6^	4 × 10^–7^	7 × 10^–7^	3 × 10^–8^
Urban aerosol	Gas-phase reactions	0	3 × 10^–5^	2 × 10^–4^	2 × 10^–4^
	Aqueous-phase reactions	1 × 10^–12^	7 × 10^–12^	1 × 10^–11^	5 × 10^–13^
		1 × 10^–11^	7 × 10^–11^	1 × 10^–10^	5 × 10^–12^

Data in Table 4 show that only in urban and remote clouds of high liquid water contents, the fluxes of 1-penten-3-ol and (*Z*)-2-hexen-1-ol removed by aqueous-phase reactions are comparable to the fluxes by gas-phase reactions. (*E*)-2-Hexen-1-al was removed faster by the gas-phase reactions in all clouds. In urban aerosol, the gas-phase removal of all GLV studied dominated the aqueous-phase one by several orders of magnitude. So was the case with all clouds.

### Atmospheric Lifetimes

The atmospheric lifetime (*t*) of a GLV removed by the gas-phase and aqueous-phase reactions with a radical *X* is the time after which the initial GLV concentration in the gas phase [GLV]_0,g_ drops to [GLV]_0,g_/e (eq 16, Section 7 in SI). The GLV and *X* partition between the phases according to Henry’s Law (Equation S22 and Figure S10 in the SI).
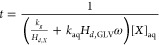
16where *k*_*g*_ and *k*_aq_ are the second-order rate constants for reactions of the GLV with *X* in the gas and aqueous phase, respectively; *H*_*d,*GLV_ and *H*_*d,X*_ are dimensionless Henry’s constants for the GLV and *X*, respectively (*H*_*d*_*= H RT*); [*X*]_aq_ is the concentration of *X* in the aqueous phase; ω is the liquid water content of the atmospheric system. Table S11 in SI shows constants required for the calculations.

**Figure 4:**
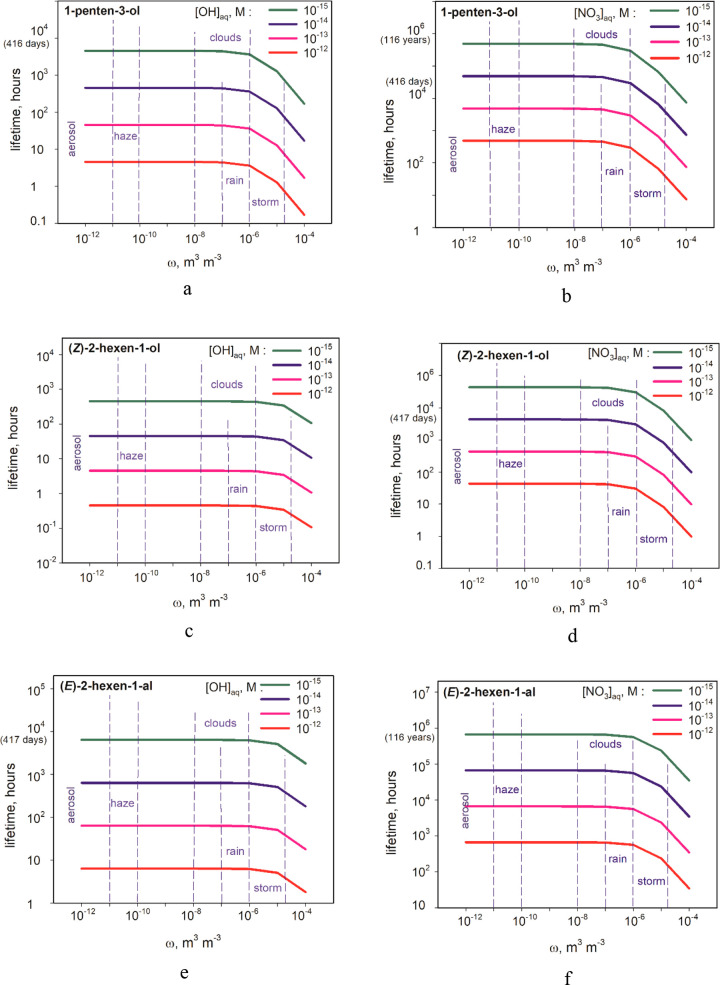
Atmospheric lifetimes (*t*) of GLVs due to combined removal by gas-phase and aqueous-phase reactions with ^•^OH (a, c, e) and NO_3_^•^ (b, d, f) at various liquid water contents (*ω*).

Equations 17 and 18 define, respectively, the lifetimes of a GLV consumed by an oxidant that exists only in the gas phase and only in the aqueous phase, e.g., SO_4_^•–^.
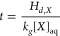
17
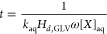
18Figure 4 shows the lifetimes of GLVs consumed by the gas-phase and the aqueous-phase reactions with ^•^OH or NO_3_^•^ radicals in the atmospheric systems with various liquid water contents (LWC). The aqueous-phase concentrations of radicals are typical (Table S12), while their gas-phase concentrations result from Henry’s equilibria. Figure S5 presents apparent lifetimes of GLVs due only to the gas-phase reactions with the radicals. The aqueous-phase reactions did not influence the lifetimes of studied GLVs in atmospheric systems with LWC < 10^–6^ (Figure 4). In systems with higher LWC, like storms, the lifetime of 1-penten-3-ol decreased significantly due to the aqueous-phase reaction with ^•^OH (Figure 4a), while the lifetimes of all GLVs studied decreased due to aqueous-phase reactions with NO_3_^•^ (Figure 4b,d,f). Figure S6 shows the atmospheric lifetime of the GLVs due to the aqueous-phase reactions with SO_4_^•–^. With increasing ω and [*X*]_aq_, the lifetimes decrease from years to hours.

### Relative GLV Removal Rates

Equation 19 compares GLV removal from the atmosphere by gas- and aqueous-phase reactions with *X* (^•^OH and NO_3_^•^) and by the aqueous-phase reactions with SO_4_^•–^.
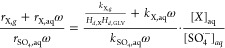
19Figure S8 shows the ratios calculated with eq 19 for various ω and radical proportions. Aqueous-phase reaction of PENTOL with SO_4_^•–^ dominates over the combined aqueous-phase and gas-phase reactions with ^•^OH radicals in clouds and rain provided SO_4_^•–^ are in excess: [^•^OH]/[SO_4_^•–^] < 0.16 (Figure S8a), and dominates over the combined reactions with NO_3_^•^ if [NO_3_^•^]/[SO_4_^•–^] < 6.5 (Figure S8b). The conditions which allow dominance of GLV reactions with SO_4_^•–^ for HEXOL are [^•^OH]/[SO_4_^•–^] < 0.40 (Figure S8c) and [NO_3_^•^]/[SO_4_^•–^] < 3 (Figure S8d); and for HEXAL – [^•^OH]/[SO_4_^•–^] < 0.11 (Figure S8e) and [NO_3_^•^]/[SO_4_^•–^] < 1 (Figure S8f). Figures S7 and S9 compare the aqueous-phase reactions of GLVs with SO_4_^•–^ with the formally separated gas-phase and aqueous-phase reactions with ^•^OH or NO_3_^•^.

## Supporting Information Available

Physical properties of the GLVs, LFP-LLPA Setup, UV Spectra of GLVs, aqueous-phase rate constants for reactions of GLVs with radicals, diffusion limitations of reactions, GLV atmospheric lifetimes and removal rates, COPASI modeling of GLV reactions with NO_3_^•^PDF)
