# Supplementary material for: Reaction Kinetics of Green Leaf Volatiles with Sulfate, Hydroxyl, and Nitrate Radicals in Tropospheric Aqueous Phase
Source: Environ Sci Technol. 2021 Sep 28;55(20):13666–76. doi: 10.1021/acs.est.1c03276 (PMC8529707; doi:10.1021/acs.est.1c03276)
Supplement: Supplementary file 1 — es1c03276_si_001.pdf [file es1c03276_si_001.pdf]

Supplementary Information for

Reaction Kinetics of Green Leaf Volatiles with Sulfate, Hydroxyl and Nitrate Radicals in Tropospheric Aqueous-Phase

Kumar Sarang[†], *Tobias Otto*[§#], *Krzysztof Rudzinski*[†*], *Thomas Schaefer*[§], *Irena Grgić*[‡], *Klara Nestorowicz*[†], *Hartmut Herrmann*[§*] *and Rafal Szmigielski*[†*]

[†] Environmental Chemistry Group, Institute of Physical Chemistry Polish Academy of Sciences, 01-224 Warsaw, Poland;

[‡]Department of Analytical Chemistry, National Institute of Chemistry, SI-1000, Ljubljana, Slovenia;

[§]Atmospheric Chemistry Department (ACD), Leibniz Institute for Tropospheric Research (TROPOS), 04318, Leipzig, Germany.

[#]now at: Labor für Wasser und Umwelt GmbH, Berliner Str. 13, 04924, Bad Liebenwerda

[*]To whom correspondence should be addressed. Email: ralf@ichf.edu.pl, kjrudz@ichf.edu.pl, phone: +48-22343-3402, Email: herrmann@tropos.de, phone: +49 341 2717 7024

Contents

1. PROPERTIES OF GLVs STUDIED S4

Table S1. Physical properties of GLVs at 298 K S4

2. LFP-LLPA EXPERIMENTAL SETUP S4

Figure S1. The Laser Flash Photolysis-Laser Long Path Absorption setup S4

3. UV SPECTROSCOPY OF GLV S4

Figure S2. UV spectra of the GLVs S5

Table S2. Molar absorption coefficients of GLVs S5

Table S3. Internal filter effect of GLV on $^{\bullet}OH$ concentration S6

4. EXPERIMENTAL DETAILS S6

Table S4. Photolysis setup for different radical generation and measurement S6

Table S5. Initial concentrations of reactants in the kinetic experiments with GLVs S7

Figure S3. Absorbance–time traces showing the decay of $SO_4^{\bullet-}$ in LFP-LLPA S7

5. ACTIVATION PARAMETERS S7

6. DIFFUSION LIMITATION OF CHEMICAL REACTIONS S8

Table S6. Properties of reacting molecules. S9

Table S7. Experimental and diffusion-corrected rate constants for reaction of GLVs with $SO_4^{\bullet-}$ S9

Table S8. Experimental and diffusion-corrected rate constants for reaction of GLVs with $^{\bullet}OH$ S10

Table S9. Experimental and diffusion-corrected rate constants for reaction of GLVs with $NO_3^{\bullet}$ S11

Figure S4. Arrhenius plots of the diffusion-corrected rate constants S12

Table S10. Diffusion-corrected activation parameters S13

7. ATMOSPHERIC SIGNIFICANCE S14

Atmospheric lifetimes, the background. S14

Table S11. Gas-phase rate constants at 298 K and Henry's constants S15

Table S12. Aqueous-phase concentrations of radicals in atmospheric systems S16

Figure S5. Lifetimes of GLVs due to the gas-phase reactions with $^{\bullet}OH$ and $NO_3^{\bullet}$. S17

Figure S6. Lifetimes of GLVs due to the aqueous-phase reactions with $SO_4^{\bullet-}$ S17

• GLV removal rates – Gas-phase vs. aqueous-phase reactions. S18

Figure S7. Gas-phase reactions with $^{\bullet}OH$ or $NO_3^{\bullet}$ radicals and aqueous-phase reactions with $SO_4^{\bullet-}$ radical-anions. S19

Figure S8. Combined gas-phase and aqueous-phase reactions $^{\bullet}OH$ or $NO_3^{\bullet}$ radicals and aqueous-phase reactions with $SO_4^{\bullet-}$ radical-anions. S20

• GLV removal rates – aqueous-phase reactions. S21

Figure S9. Aqueous-phase reaction with $^{\bullet}OH$ or $NO_3^{\bullet}$ vs. reactions with $SO_4^{\bullet-}$ S21

• GLV partitioning between gas and aqueous phases. S22

Figure S10. Gas-aqueous partitioning of GLV in various atmospheric systems S22

8. BIAS OF THE EXPERIMENTAL RATE CONSTANTS FOR $NO_3^{\bullet}$ REACTIONS WITH GLV S22

Table S13. Model_1 – reactions with temperature-dependent rate constants S23-24

• Kinetic parameters for reactions (13), (15), (24), and (25) S25

Table S14. The temperature-dependent rate constants for reaction (13) S25

Figure S11. Arrhenius plot for reaction (13) $SO_4^{\bullet-} + NO_3^{-} \longrightarrow NO_3^{\bullet} + SO_4^{2-}$ S26

Table S15. The temperature-dependent rate constants for reaction (15). S26

Figure S12. Arrhenius plot for reaction (15) $SO_4^{\bullet-} + S_2O_8^{2-} \longrightarrow S_2O_8^{\bullet-} + SO_4^{2-}$ S27

Table S16. The temperature-dependent rate constants for reaction (24) S27

Figure S13. Arrhenius plot for reaction (24) $NO_3^{\bullet} + S_2O_8^{2-} \longrightarrow NO_3^{-} + S_2O_8^{\bullet-}$ S28

Table S17. The estimation of the rate constants for a first-order sink of $NO_3^{\bullet}$ S28

• Estimation of GLV + $NO_3^{\bullet}$ rate constants at various temperatures S29

Figures S14-S26. Experimental and simulated concentration-time-profiles of $NO_3^{\bullet}$ S30

9. REFERENCES S42

▪ **1. PROPERTIES OF GLVs STUDIED**

Table S1. Physical properties of selected GLVs at 298 K estimated with EPI suite[1]

**GLV**	**Molecular formula**	**The Henry's Law Constant for water**	**Vapor pressure**	**Water solubility**
		mol L^{-1} atm^{-1}	atm	mg L^{-1}
**1-penten-3-ol**	$C_5H_{10}O$	101.22	1.20×10^{-2}	4.53×10^{4}
**(Z)-2-hexen-1-ol**	$C_6H_{12}O$	133.26	1.19×10^{-3}	1.60×10^{4}
**(E)-2-hexen-1-al**	$C_6H_{10}O$	10.12	6.21×10^{-3}	5.26×10^{3}

▪ **2. LFP-LLPA EXPERIMENTAL SETUP**

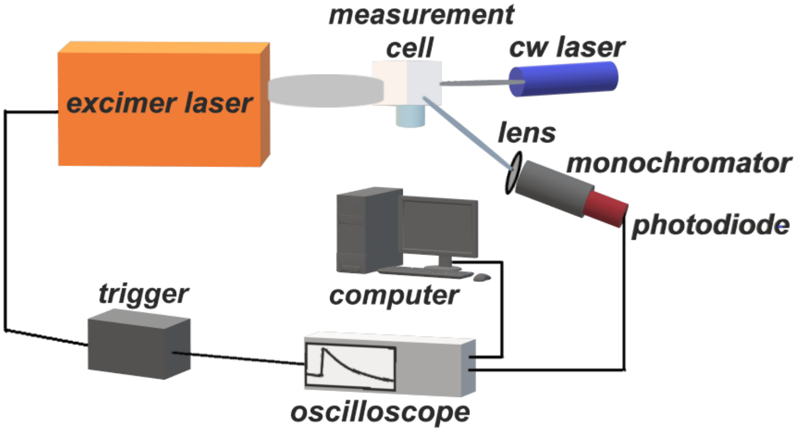

Figure S1. The Laser Flash Photolysis-Laser Long Path Absorption (LFP-LLPA) setup used for the kinetic investigations.

▪ **3. UV SPECTROSCOPY OF GLV**

The molar absorption coefficients of GLVs were determined from absorption spectra measured with a Lambda 900 UV/VIS/NIR spectrometer (Perkin Elmer Instruments). The UV spectra were recorded over the wavelength range of 200 – 400 nm and for the concentrations ranging from 500 – 31.25 mM (PENTOL); 100 – 6.25 mM (HEXOL); 30 – 1.875 mM (HEXAL). Molar absorption coefficients were determined at 248, 308, and 351 nm using the average of experimental molar extinction coefficients at each concentration point. For (E)-2-hexen-1-al, the absorbance could be

recorded only at wavelengths longer than 245 nm. The uncertainties of the coefficients were equal Vto the standard deviations (shown as yellow ranges).

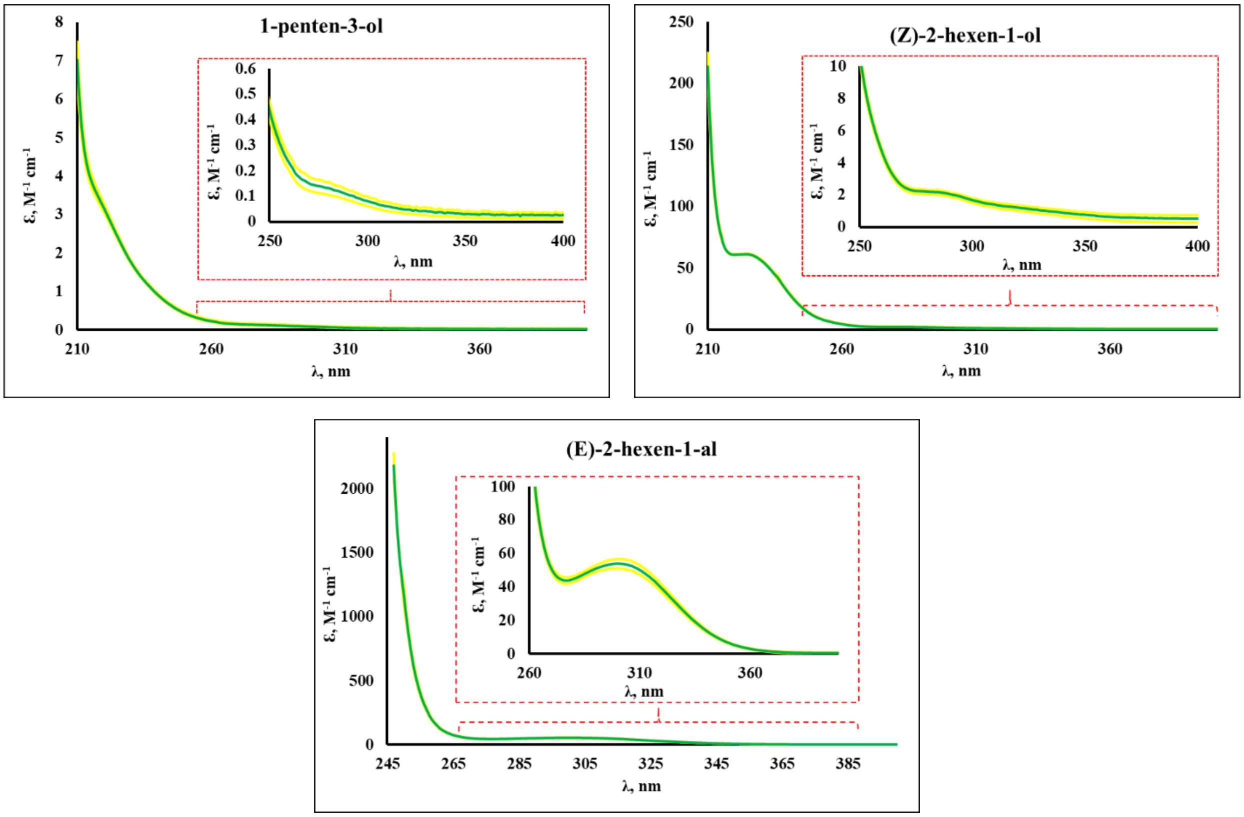

Figure S2. UV spectra of 1-penten-3-ol, (Z)-2-hexen-1-ol, and (E)-2-hexen-1-al.

Table S2 shows the molar absorption coefficients' values at three wavelengths, which were used to correct the internal filter effects (explained in the main text) due to GLV reactants in experiments with $^{\bullet}OH$ radicals (Table S3).

Table S2. Selected numerical values of the molar absorption coefficients of the examined GLVs used to calculate the filter effects in Table S3.

**Wavelength**	ε		
**nm**	L mol^{-1} cm^{-1}		
	1-penten-3-ol	(Z)-2-hexen-1-ol	(E)-2-hexen-1-al
**248**	0.51 ± 0.03	13.3 ± 0.4	1722 ± 13
**308**	0.06 ± 0.02	1.4 ± 0.2	51.8 ± 2.8
**351**	0.03 ± 0.01	0.7 ± 0.2	6.4 ± 0.3

Table S3. Change in the initial $^{\bullet}OH$ concentrations due to a GLV as an internal filter of the UV light.

Experiment	[GLV]	$[OH]_0$ change, %		
	10^{-4} L mol^{-1}	1-penten-3-ol at 248 nm	(Z)-2-hexen-1-ol at 248 nm	(E)-2-hexen-1-al at 308 nm
**I**	0.5	0.01	0.23	0.87
**II**	1.0	0.02	0.45	1.74
**III**	1.5	0.03	0.68	2.59
**IV**	2.0	0.03	0.90	3.44

- ## 4. EXPERIMENTAL DETAILS

Table S4. Photolysis setup for different radical generation and measurement

Radical	GLV	Radical precursor	Excimer laser	Continuous-wave laser	Total path length
			λ, nm	λ, nm	cm
$SO_4^{\bullet -}$	1-penten-3-ol	$Na_2S_2O_8$	248	407	32
	(Z)-2-hexen-1-ol		248	407	
	(E)-2-hexen-1-al		308	473	
$^{\bullet}OH$	1-penten-3-ol	H_2O_2	248	407	32
	(Z)-2-hexen-1-ol		248	407	
	(E)-2-hexen-1-al		308	473	
$NO_3^{\bullet}$	1-penten-3-ol	$NaNO_3$	351	635	128
	(Z)-2-hexen-1-ol				
	(E)-2-hexen-1-al				

Table S5: Initial concentrations of reactants (mM) in the kinetic experiments with GLVs.

GLV	[GLV]	$SO_4^{\bullet-}$	$^{\bullet}OH$		NO_3	
		$Na_2S_2O_8$	H_2O_2	KSCN	$NaNO_3$	$Na_2S_2O_8$
**1-penten-3-ol**	0.005 – 0.2	0.5	0.2	0.02	100	30
**(Z)-2-hexen-1-ol**	0.01 – 0.2	0.5	0.2	0.02	100	30
**(E)-2-hexen-1-al**	0.0025 – 0.2	5.0	10	0.20	100	30

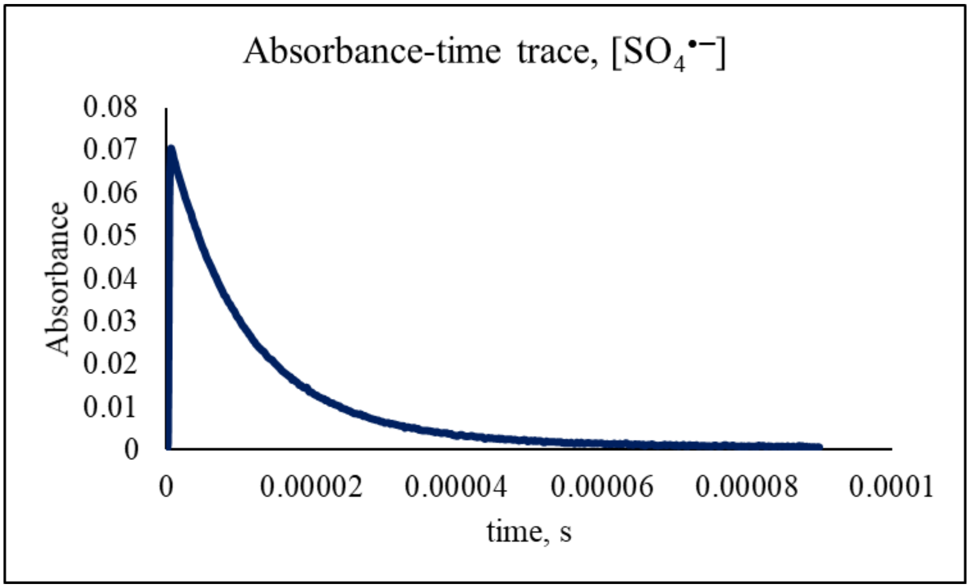

Figure S3. Typical absorbance–time traces observed following a laser flash photolysis. Here shown as an example from $Na_2S_2O_8$ (0.5 mM) and 1-penten-3-ol (0.1 mM) mixture in the water at 298 K showing the rapid decay of the sulfate radical-ion.

▪ 5. ACTIVATION PARAMETERS

Calculation of activation parameters is described in detail elsewhere. [2-4] The activation parameters were calculated using the equations (S1-S4).

Arrhenius equation: $$k(T) = A\, exp \frac{-E_A}{RT} \quad (S1)$$

Enthalpy of activation: $$\Delta H^{\ddagger} = E_A - RT \quad (S2)$$

Entropy of activation: $$\Delta S^{\ddagger} = R\left[\ln A - \ln \frac{k_B T}{h} - 1\right] \quad (S3)$$

Gibb's free energy of activation: $$\Delta G^{\ddagger} = \Delta H^{\ddagger} - T\Delta S^{\ddagger} \quad (S4)$$

where: Boltzmann constant $k_B = 1.38 \times 10^{23}\, JK^{-1}$, Planck constant $h = 6.626 \times 10^{-34}\, Js$.

6. DIFFUSIONAL LIMITATION OF CHEMICAL REACTIONS

An experimentally observed second-order rate constant that is limited by the diffusion of reactants, k_{obs}, can be split into the true rate constant k_{reac} and the diffusion rate constant k_{diff} using a simple resistance-in-series approach:

$$k_{obs}^{-1} = k_{reac}^{-1} + k_{diff}^{-1} \quad \text{(S5)}$$

The diffusion rate constant is calculated using the Smoluchowski Equation (S11):[5]

$$k_{diff} = 4 \times 10^3 \pi N_A (D_A + D_B)(r_A + r_B) \quad \text{(S6)}$$

where: N_A is the Avogadro number, D_A and D_B ($m^2\ s^{-1}$) are the diffusion coefficients of reactants, r_A and r_B (m) are reaction radii of the reactants.

The diffusion coefficients are calculated using the Stoke-Einstein relationship modified by Wilke and Chang: [6]

$$D = 7.4 \times 10^{-12} \frac{(XM)^{0.5} T}{{V_m}^{0.6} \eta} \quad \text{(S7)}$$

where: X is the association parameter of a solvent (2.26 for water), M is the molar mass of a diffusing compound, $cm^3\ mol^{-1}$, T is the absolute temperature in K, V_m is the molar volume of a diffusing compound[7], η is the dynamic viscosity of the solvent, mPa or 0.01 $g\ cm^{-1}\ s^{-1}$ (0.8891 mPa for water at 298 K). Molar volumes for reactants in the present work are given in Table S8.

The reaction radii of the reacting compounds were calculated using the procedure of Kojima and Bard (1975) [8]

$$r = \sqrt[3]{\frac{3 \times V_m}{4 \times \pi \times N_A}} \quad \text{(S8)}$$

The radical radii were adopted from Buxton et al., 1988 ($^{\bullet}OH$),[9] and Nightingale et al., 1959 ($SO_4^{\bullet-}$ & $NO_3^{\bullet}$).[10] The radical molar volumes were adopted from Schöne et al. 2014,[3] whereas the molar volumes of the organic compounds were calculated using Tyn and Claus method (1975) given in equation (S9)[11] and critical volume described by Joback and Reid, 1987 (equation S10)[7] at the boiling point. The final molar volume at measurement temperatures were obtained using the ideal gas approach (V_m/T= constant).

$$V_m = 0.285 \times V_C^{1.048} \quad \text{(S9)}$$

$$V_C = 17.5 + \sum \Delta V \quad \text{(S10)}$$

Table S6: Properties of reacting molecules.

Molecule	V_m	r	D (at 298 K)
	$cm^3\ mol^{-1}$	nm	$10^{-9}\ m^2\ s^{-1}$
**$SO_4^{\bullet-}$**	61.5[a]	0.29[a]	1.30
**$^{\bullet}OH$**	26.9[a]	0.22[a]	2.20
**$NO_3^{\bullet}$**	46.4[a]	0.264[a]	1.54
**1-penten-3-ol**	89.4[b]	0.329	1.06
**(Z)-2-hexen-1-ol**	95.2[b]	0.335	1.03
**(E)-2-hexen-1-al**	99.2[b]	0.340	1.00

a Schöne et al.[3]; **b** Estimated using Joback method.[7]

Table S7. Comparison of experimentally observed and diffusion-corrected rate constants for reactions of GLVs with $SO_4^{\bullet-}$, and rate constants for the diffusion of reactants (k_{obs}, k_{reac}, *and* k_{diff}, respectively, 10^8 L mol^{-1} s^{-1}).

GLV + $SO_4^{\bullet-}$		278 K	288 K	298 K	308 K	318 K
**PENTOL**	k_{obs}	8.2 ± 0.7	9.4 ± 0.7	9.4 ± 1.0	10.5 ± 2.1	11.0 ± 2.4
	k_{reac}	9.0 ± 0.8	10.4 ± 0.8	10.2 ± 1.1	11.5 ± 2.3	12.1 ± 2.7
	k_{diff}	88.2	98.8	111	124	126
	% k_{diff}	9	10	9	9	9
**HEXOL**	k_{obs}	17.1 ± 2.0	22.0 ± 3.3	25.3 ± 3	28.1 ± 6.5	28.7 ± 6.3
	k_{reac}	21.2 ± 2.5	28.4 ± 4.2	32.9 ± 3.9	36.4 ± 8.5	37.3 ± 8.2
	k_{diff}	88.1	98.4	110	123	125
	% k_{diff}	19	22	23	23	23
**HEXAL**	k_{obs}	4.2 ± 0.2	4.5 ± 0.1	4.8 ± 0.2	5.3 ± 0.3	5.1 ± 0.5
	k_{react}	4.4 ± 0.2	4.7 ± 0.1	5.0 ± 0.2	5.6 ± 0.3	5.4 ± 0.5
	k_{diff}	88.1	98.3	110	123	124
	% k_{diff}	5	5	4	4	4

Table S8. Comparison of experimentally observed and diffusion-corrected rate constants for reactions of GLVs with $^{\bullet}OH$, and rate constants for the diffusion of reactants (k_{obs}, k_{reac}, and k_{diff}, respectively, 10^9 L mol^{-1} s^{-1}).

GLV + $^{\bullet}$OH		278 K	288 K	298 K	308 K	318 K
**PENTOL**	k_{obs}	4.01 ± 0.04	5.1 ± 0.2	6.3 ± 0.1	6.5 ± 0.3	8.5 ± 1.0
	k_{reac}	6.2 ± 0.1	8.7 ± 0.3	11.8 ± 0.3	11.5 ± 0.6	19.8 ± 2.4
	k_{diff}	11.5	12.5	13.6	14.8	14.9
	% k_{diff}	35	41	46	44	57
**HEXOL**	k_{obs}	4.49 ± 0.04	5.4 ± 0.2	6.7 ± 0.3	6.3 ± 0.3	7.8 ± 0.4
	k_{reac}	7.4 ± 0.1	9.5 ± 0.3	13.1 ± 0.6	11.0 ± 0.4	16.4 ± 0.8
	k_{diff}	11.5	12.5	13.6	14.8	14.9
	% k_{diff}	39	43	49	43	52
**HEXAL**	k_{obs}	3.5 ± 0.1	4.6 ± 0.1	4.8 ± 0.3	6.0 ± 0.3	6.6 ± 0.4
	k_{reac}	4.9 ± 0.2	7.4 ± 0.2	7.4 ± 0.5	10.1 ± 0.5	12.0 ± 0.7
	k_{diff}	11.6	12.5	13.6	14.7	14.9
	% k_{diff}	30	37	35	41	45

Table S9. Comparison of experimentally observed and diffusion-corrected rate constants for reactions of GLVs with NO_3, and rate constants for the diffusion of reactants (k_{obs}, k_{reac}, and k_{diff}, respectively, 10^7 L mol^{-1} s^{-1}).

GLV + $NO_3^•$		278 K	288 K	298 K	308 K	318 K
PENTOL	k_{obs}	8.8 ± 1.6	10.5 ± 2.3	15.0 ± 1.5	19.8 ± 4.2	21 ± 9
	k_{reac}	8.9 ± 1.6	10.6 ± 2.3	15.2 ± 1.5	20.1 ± 4.3	20.6 ± 9.2
	k_{diff}	950	1053	1169	1297	1313
	% k_{diff}	1	1	1	2	2
HEXOL	k_{obs}	64.3 ± 14.8	79.1 ± 24.3	83.7 ± 22.8	97.7 ± 39.3	83 ± 19*
	k_{reac}	69 ± 16	85.5 ± 26.2	90.2 ± 24.6	105.7 ± 42.5	-
	k_{diff}	951	1051	1165	1290	-
	% k_{diff}	7	8	7	8	-
HEXAL	k_{obs}	2.0 ± 0.6	2.1 ± 0.2	3.0 ± 0.7	3.7 ± 0.1	5.0 ± 0.8
	k_{reac}	2.0 ± 0.6	2.2 ± 0.2	3.0 ± 0.7	3.7 ± 0.1	5.0 ± 0.8
	k_{diff}	952	1050	1160	1290	1300
	% k_{diff}	0.2	0.2	0.3	0.3	0.4

* at T = 293 K

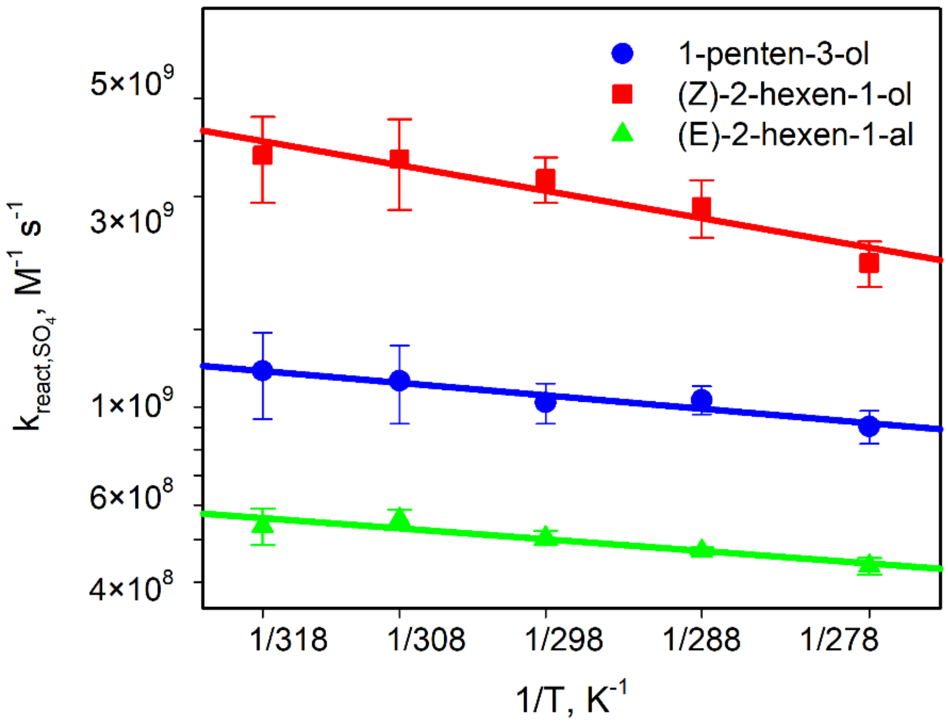

Figure S4a. Arrhenius plots of the rate constants corrected for diffusional limitations, k_{reac} for the reaction of GLVs with $SO_4^{\bullet-}$ (see Table S7 for the numerical values of the constants).

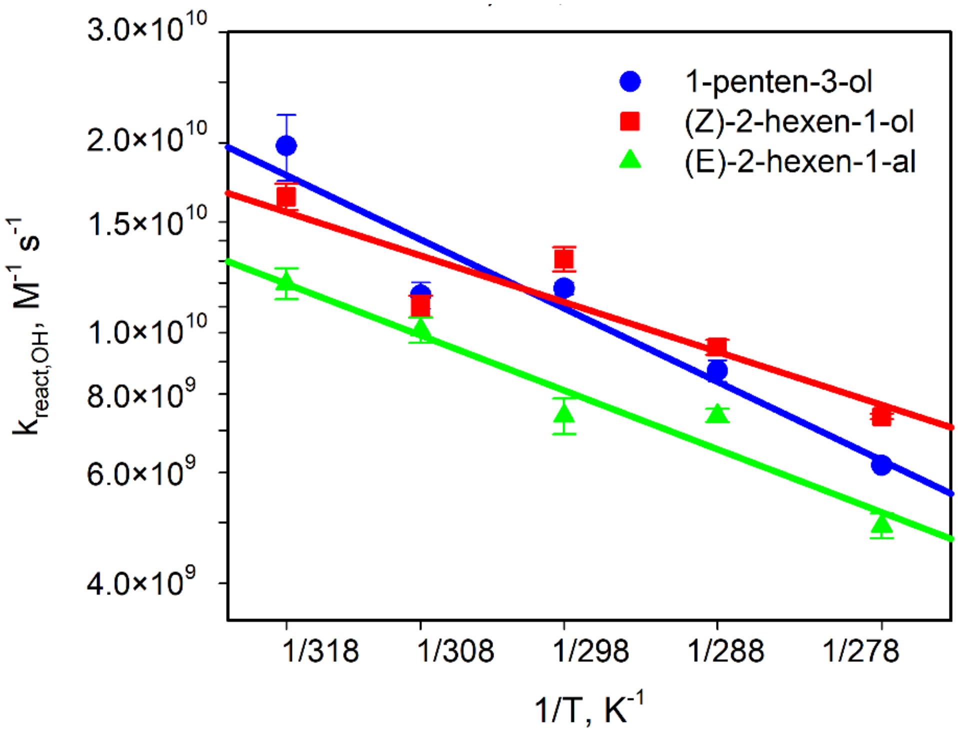

Figure S4b. Arrhenius plots of the rate constants corrected for diffusional limitations, k_{reac} for the reaction of GLVs with $^{\bullet}OH$ (see Table S8 for the numerical values of the constants).

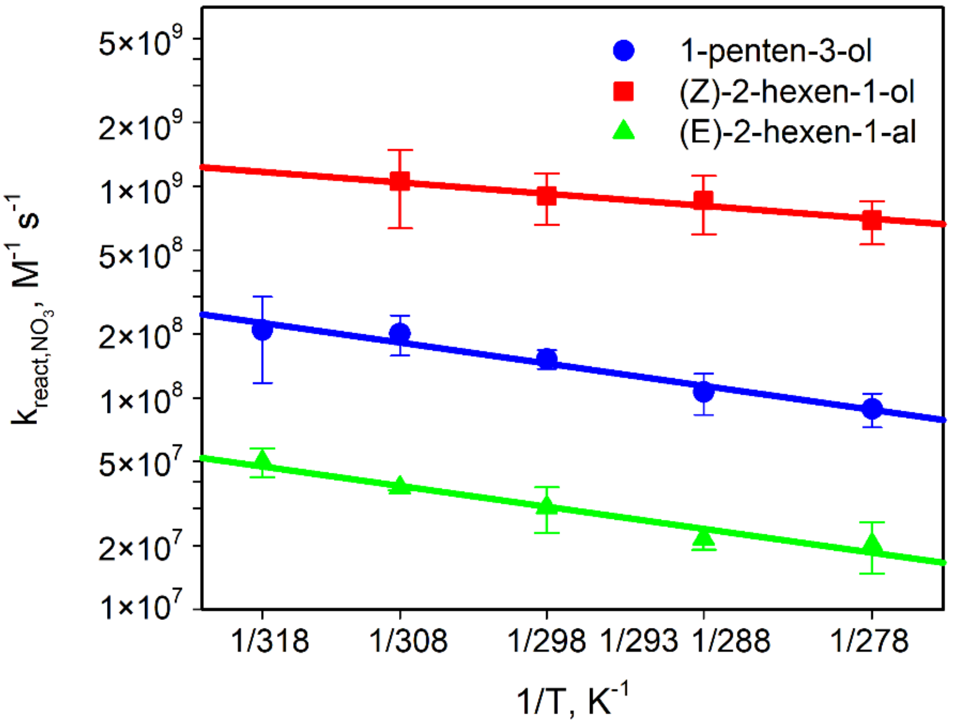

Figure S4c. Arrhenius plots of the rate constants corrected for diffusional limitations, k_{reac} for the reaction of GLVs with $NO_3^{•}$ (see Table S9 for the numerical values of the constants).

Table S10. The diffusion-corrected activation parameters for the reactions of GLVs with $SO_4^{•-}$, $^{•}OH$ and $NO_3^{•}$ radicals.

Reactants		E_A	A	$\Delta H^{\ddagger}$	$-\Delta S^{\ddagger}$	$\Delta G^{\ddagger}$
		kJ mol^{-1}	L mol^{-1} s^{-1}	kJ mol^{-1}	J mol^{-1} K^{-1}	kJ mol^{-1}
	PENTOL	5 ± 1	(8.0 ± 0.1)×10^{9}	3 ± 1	64 ± 1	22 ± 5
**$SO_4^{•-}$**	HEXOL	10 ± 2	(19 ± 1)×10^{10}	8 ± 2	37 ± 1	19 ± 5
	HEXAL	4 ± 1	(2.9 ± 0.1)×10^{9}	2 ± 1	72 ± 2	23 ± 6
	PENTOL	19 ± 3	(2.5 ± 0.1)×10^{13}	17 ± 4	-3.2 ± 0.2	16 ± 4
**$^{•}OH$**	HEXOL	13 ± 3	(2.1 ± 0.1)×10^{12}	11 ± 3	17 ± 1	16 ± 6
	HEXAL	15 ± 2	(3.9 ± 0.2)×10^{12}	13 ± 2	12 ± 1	17 ± 4
	PENTOL	17 ± 2	(1.6 ± 0.1)×10^{11}	15 ± 2	39 ± 2	26 ± 5
**$NO_3^{•}$**	HEXOL	9 ± 1	(4 ± 1)×10^{10}	7 ± 1	50 ± 1	22 ± 4
	HEXAL	17 ± 2	(3.2 ± 0.1)×10^{10}	15 ± 2	52 ± 2	30 ± 6

▪ 7. ATMOSPHERIC SIGNIFICANCE

Atmospheric lifetimes, the background. Washing a GLV from the atmosphere's gas phase by combined gas-phase and aqueous-phase reactions with a radical *X* is approximately described by Equation (S11).

$$\frac{d[GLV]_g}{dt} = -\left(k_g[X]_g[GLV]_g + k_{aq}[X]_{aq}[GLV]_{aq}\omega\right) \tag{S11}$$

where: k_g *and* k_{aq}, L mol^{-1} s^{-1} are the second-order rate constants for reactions of the GLV with *X* in the gas and aqueous phase, respectively; ω, m^3 m^{-3} is the liquid water contents of the atmospheric system; $[\ ]_g$ and $[\ ]_{aq}$, M are the concentrations in the gas- and aqueous phases, respectively.
We assume that the gas- and aqueous forms of the reactants are bound by Henry's Law equilibria (S12) and (S13).

$$[GLV]_{aq} = H_{d,GLV}[GLV]_g \tag{S12}$$

$$[X]_g = {[X]_{aq}}/{H_{d,X}} \tag{S13}$$

with dimensionless Henry's constants H_d defined by Equations (S14).

$$H_d = HRT,\ if\ H\ is\ in\ \mathrm{mol\ L^{-1}atm^{-1}} \tag{S14a}$$

$$H_d = HRT\rho,\ if\ H\ is\ in\ \mathrm{mol\ kg^{-1}atm^{-1}} \tag{S14b}$$

where: *R*, atm L mol^{-1} K^{-1} is the gas constant; ρ kg L^{-1} is the density of solution. Equations (S11) - (S13) are combined and rearranged to Equation (S15).

$$\frac{d[GLV]_g}{[GLV]_g} = -\left(\frac{k_g}{H_{d,X}} + k_{aq}H_{d,GLV}\omega\right)[X]_{aq}dt \tag{S15}$$

Equation (S18) is integrated within the borders (t, $[GLV]_0$) and (t, $[GLV]$) to give (S16).

$$ln\left(\frac{[GLV]_g}{[GLV]_{g,0}}\right) = -\left(\frac{k_g}{H_{d,X}} + k_{aq}H_{d,GLV}\omega\right)[X]_{aq}t \tag{S16a}$$

or $$\frac{[GLV]_g}{[GLV]_{g,0}} = exp\left(-\left(\frac{k_g}{H_{d,X}} + k_{aq}H_{d,GLV}\omega\right)[X]_{aq}t\right) \tag{S16b}$$

Now, we define the lifetime of a GLV, t_{life}, as the time in which the concentration of this GLV decreases to $[GLV]_0/e$. We insert this value to Equation (S16b) to get (S17).

$$\frac{1}{e} = exp\left(-\left(\frac{k_g}{H_{d,X}} + k_{aq}H_{d,GLV}\omega\right)[X]_{aq}t_{life}\right) \tag{S17a}$$

or $$t_{life} = \frac{1}{\left(\frac{k_g}{H_{d,X}} + k_{aq} H_{d,GLV} \omega\right)[X]_{aq}} \quad \text{(S17b)}$$

Per analogiam, the lifetime of a GLV consumed by a reaction with a radical *X,* which exists only in the aqueous-phase and does not partition to the gas phase, is given by Equation (S18).

$$t_{life,aq} = \frac{1}{k_{aq} H_{d,GLV} \omega [X]_{aq}} \quad \text{(S18)}$$

When a GLV disappears solely by the reaction with a radical *X*, which does not partition to the aqueous phase, its lifetime is given by Equation (S19).

$$t_{life,g} = \frac{H_{d,X}}{k_g [X]_{aq}} \quad \text{(S19)}$$

If a GLV is removed from the atmosphere by the combined gas-phase and aqueous-phase reactions, and one wants to separate those processes virtually, equations (S18) and (S19) help. Table S11 contains the constants required to apply the above equations to GLVs studied in this work.

Table S11. Gas-phase rate constants of GLVs with atmospheric radicals at 298 K and dimensionless Henry's constant of GLVs and radicals.

GLV/radical	Gas-phase rate constants		Gas-phase rate constants		H_d
	cm^3 $molecule^{-1}$ s^{-1}		$L\ mol^{-1}\ s^{-1}$		
	k_{OH}	k_{NO3}	k_{OH}	k_{NO3}	
**PENTOL**	6.7×10^{-11} [12]<br>5.7×10^{-11} [13]	13.9×10^{-15} [14]	3.7×10^{10}	8.4×10^{6}	2.5×10^{3}
**HEXOL**	6.2×10^{-11} [1]	1.6×10^{-13} [15]	3.7×10^{11}	9.4×10^{7}	3.3×10^{3}
**HEXAL**	4.4×10^{-11} [16]	1.2×10^{-14} [16]	2.6×10^{11}	6.1×10^{6}	2.5×10^{2}
**$^{\bullet}OH$**					6.1×10^{2}
**$NO_3^{\bullet}$**					14.7

*originally, the gas phase rate constants were reported in cm^3 $molecule^{-1}$ s^{-1}, and were converted to $L\ mol^{-1}\ s^{-1}$ for the convenience of present calculations.

Table S12. Aqueous-phase concentrations of $^{\bullet}OH$, $NO_3^{\bullet}$ and $SO_4^{\bullet-}$ radicals in various atmospheric systems from CAPRAM modeling.[17]

**Radical**	**low**		**high**		**extremely high**	
	mol L^{-1}		mol L^{-1}		mol L^{-1}	
**$^{\bullet}OH$**	urban clouds	3.5×10^{-15}	maritime aerosol	1.0×10^{-13}	maritime clouds	2.0×10^{-12}
	remote clouds	2.2×10^{-14}	urban aerosol	4.4×10^{-13}	remote aerosol	3.0×10^{-12}
**$NO_3^{\bullet}$**	remote clouds	5.1×10^{-15}	remote aerosol	3.5×10^{-13}		
	maritime aerosol	1.9×10^{-15}	urban clouds	1.4×10^{-13}		
	maritime clouds	6.9×10^{-15}	urban aerosol	8.6×10^{-14}		
**$SO_4^{\bullet-}$**	maritime clouds	2.3×10^{-15}	remote clouds	2.4×10^{-14}	remote aerosol	3.6×10^{-13}
	urban aerosol	9.3×10^{-15}	maritime aerosol	1.2×10^{-14}		
			urban clouds	1.1×10^{-14}		

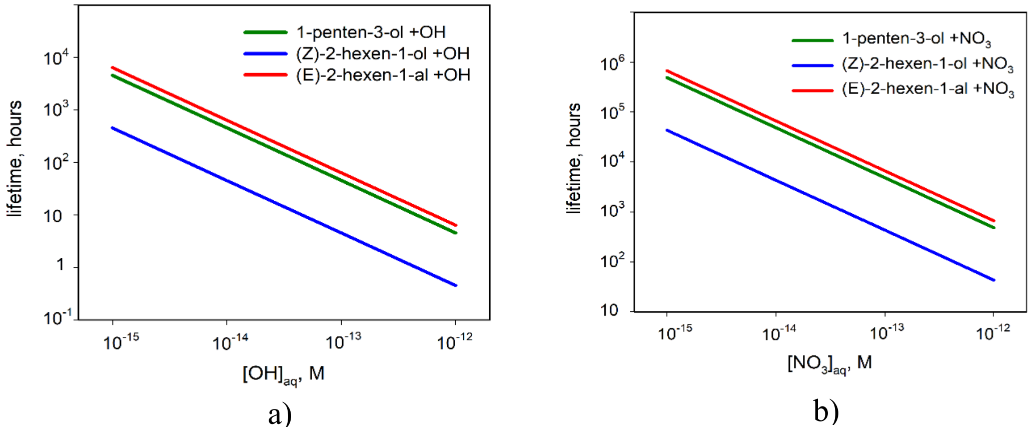

Figure S5. Apparent lifetimes of GLVs due only to the gas-phase reactions with (a) $^{\bullet}OH$ and (b) $NO_3^{\bullet}$.

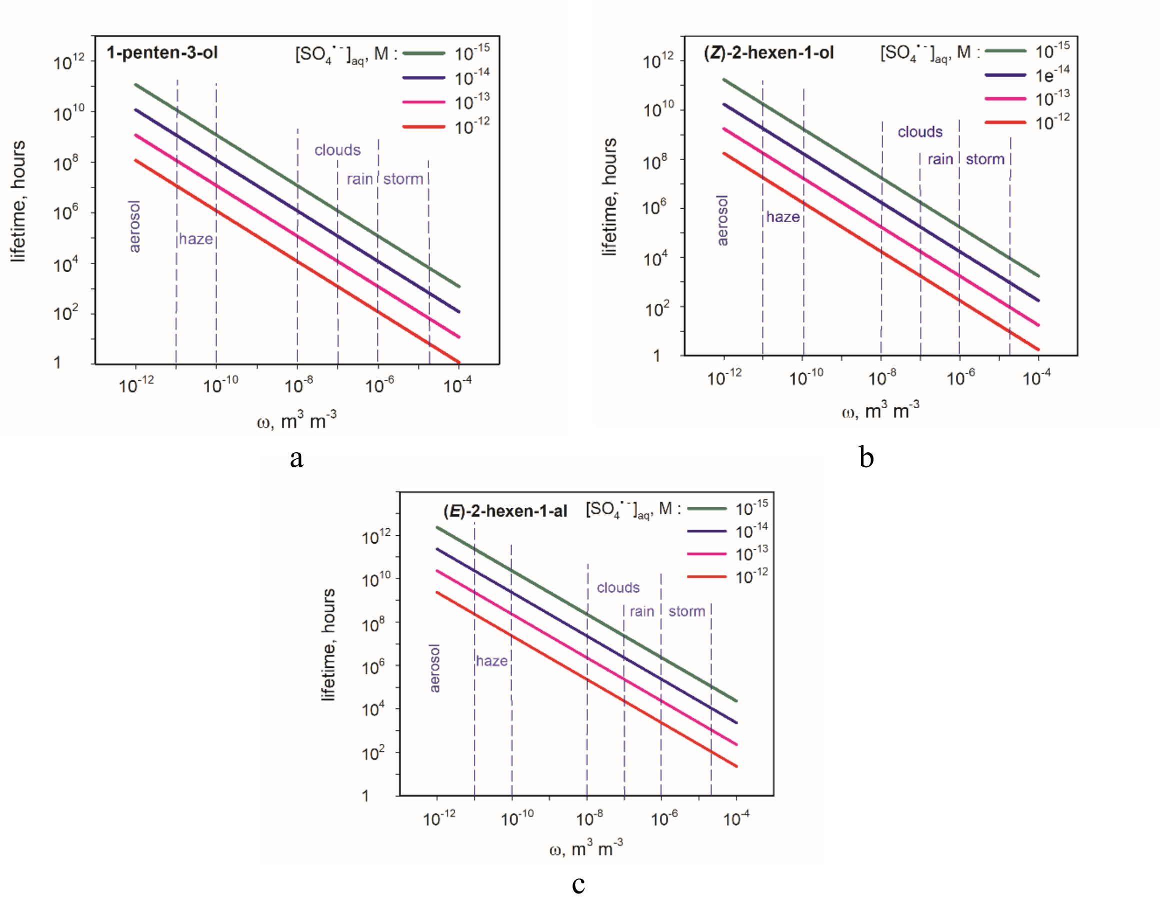

Figure S6. Atmospheric lifetimes of GLVs due to the aqueous-phase reactions with $SO_4^{\bullet-}$ at various liquid water contents (ω): (a) PENTOL, (b) HEXOL, (c) HEXAL.

GLV removal rates – Gas-phase vs. aqueous-phase reactions. Equation (S20) compares a GLV removal from the atmosphere by a gas-phase reaction with a radical *X* with that GLV removal by the aqueous-phase reaction with $SO_4^{\bullet -}$.

$$\frac{r_{X,g}}{r_{SO_4,aq}\omega} = \frac{k_{X,g}}{k_{SO_4,aq}H_{d,X}H_{d,GLV}\omega} \cdot \frac{[X]_{aq}}{[SO_4^{\cdot -}]_{aq}} \tag{S20}$$

Figure (S7) shows the ratios calculated with Equation (S20) for various ω and proportions of radicals. Aqueous-phase reaction of $SO_4^{\bullet -}$ with PENTOL dominates over the gas-phase reaction with $^{\bullet}OH$ in systems with high liquid water contents provided that $SO_4^{\bullet -}$ is in excess: $[^{\bullet}OH]/[SO_4^{\bullet -}] < 0.2$, and dominates in clouds and rains over the gas-phase reaction with $NO_3^{\bullet}$ if: $[NO_3^{\bullet}]/[SO_4^{\bullet -}] < 20$. The conditions which secure a similar dominance of GLV reactions with $SO_4^{\bullet -}$ for HEXOL are: $[^{\bullet}OH]/[SO_4^{\bullet -}] < 0.01$ and $[NO_3^{\bullet}]/[SO_4^{\bullet -}] < 10$; and for HEXAL – $[^{\bullet}OH]/[SO_4^{\bullet -}] < 0.01$ and $[NO_3^{\bullet}]/[SO_4^{\bullet -}] < 1$.

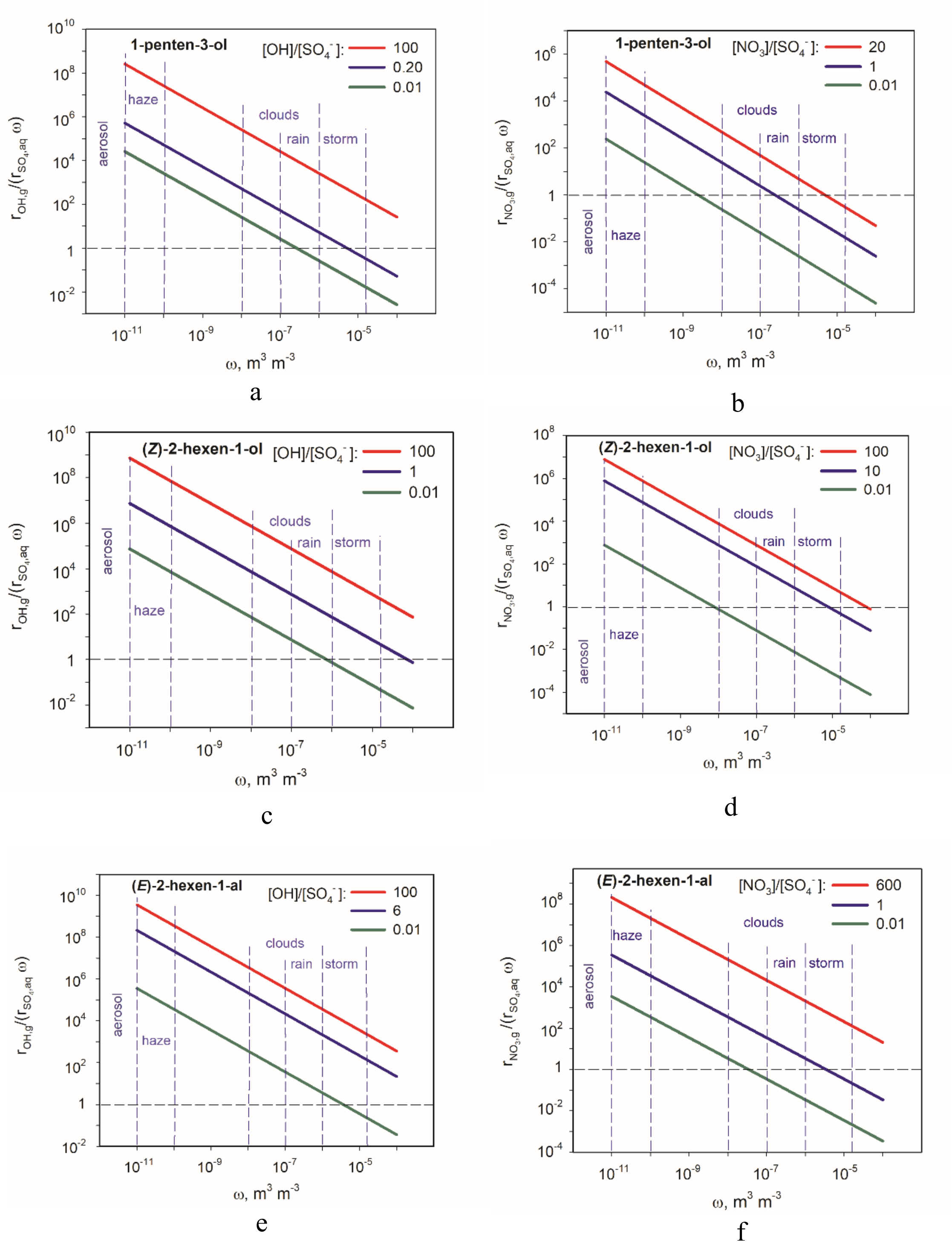

Figure S7. Influence of the liquid water contents (ω) and radical ratios on the relative rates of GLV removal from the atmosphere by gas-phase reactions with $^{\bullet}OH$ (a,c,e) or $NO_3^{\bullet}$ (b,d,f) and by the aqueous-phase reactions with $SO_4^{\bullet-}$ defined by Equation (S20).

Equation (19) in the main text compares GLV removal from the atmosphere by gas- and aqueous-phase reactions with X ($^{\bullet}OH$ and $NO_3^{\bullet}$) and by the aqueous-phase reactions with $SO_4^{\bullet-}$. Figure S8 shows the ratios calculated with that equation.

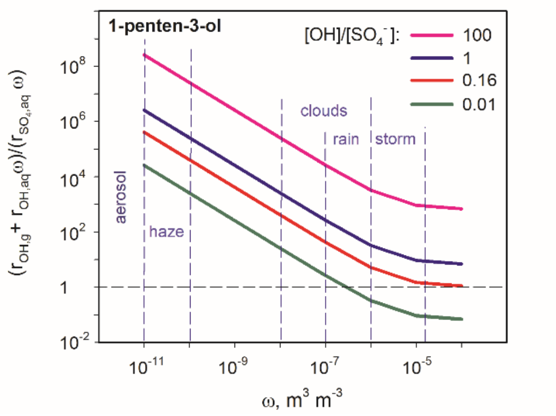

a

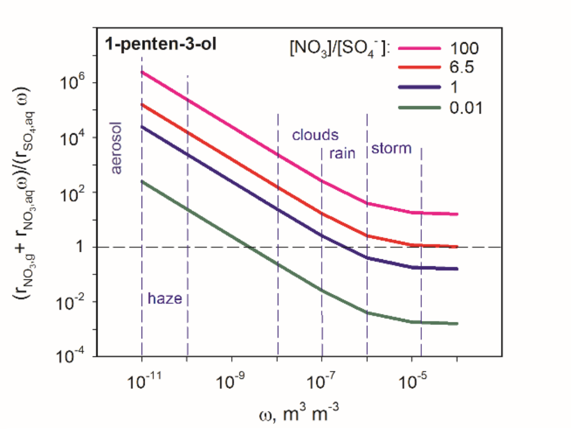

b

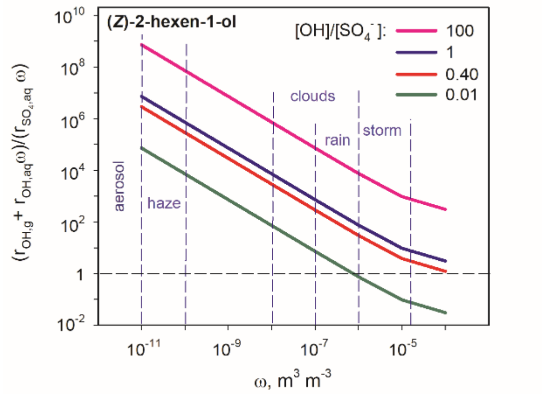

c

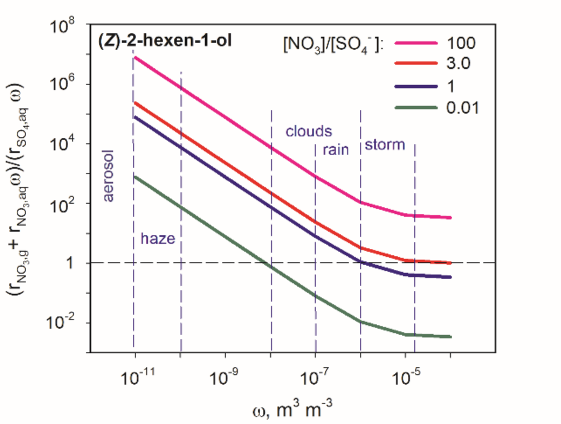

d

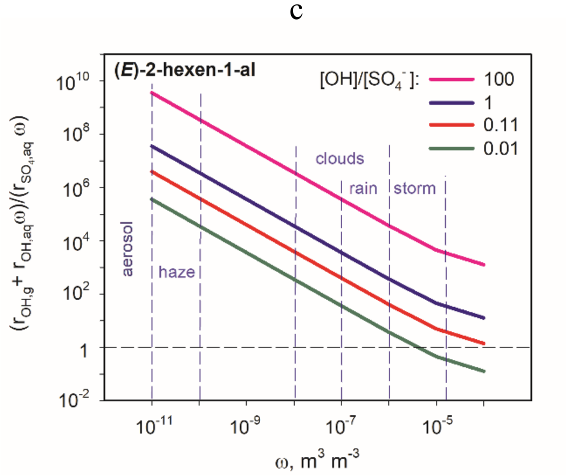

e

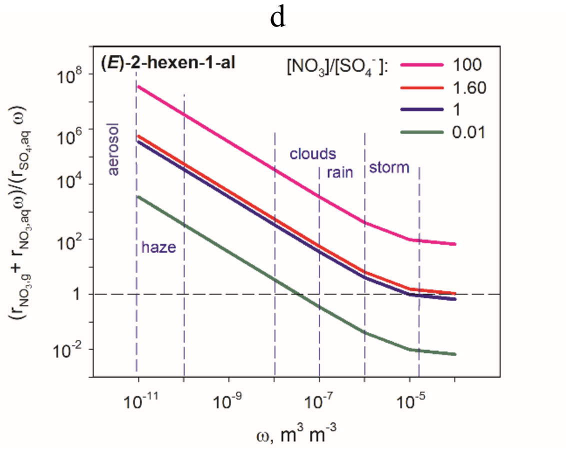

f

Figure S8. Influence of the liquid water contents (ω) and radical ratios on the relative rates of GLV removal from the atmosphere by combined gas-phase and aqueous-phase reactions with $^{\bullet}OH$ (a,c,e) or $NO_3^{\bullet}$ (b,d,f) and by the aqueous-phase reactions with $SO_4^{\bullet -}$.

GLV removal rates – aqueous-phase reactions. Equation (S21) compares a GLV removal from the atmosphere by an aqueous-phase reaction with a radical *X* with that GLV removal by the aqueous-phase reaction with $SO_4^{\bullet -}$.

$$\frac{r_{X,aq}}{r_{SO_4,aq}} = \frac{k_{X,aq}}{k_{SO_4,aq}} \cdot \frac{[X]_{aq}}{[SO_4^{-}]_{aq}} \tag{S21}$$

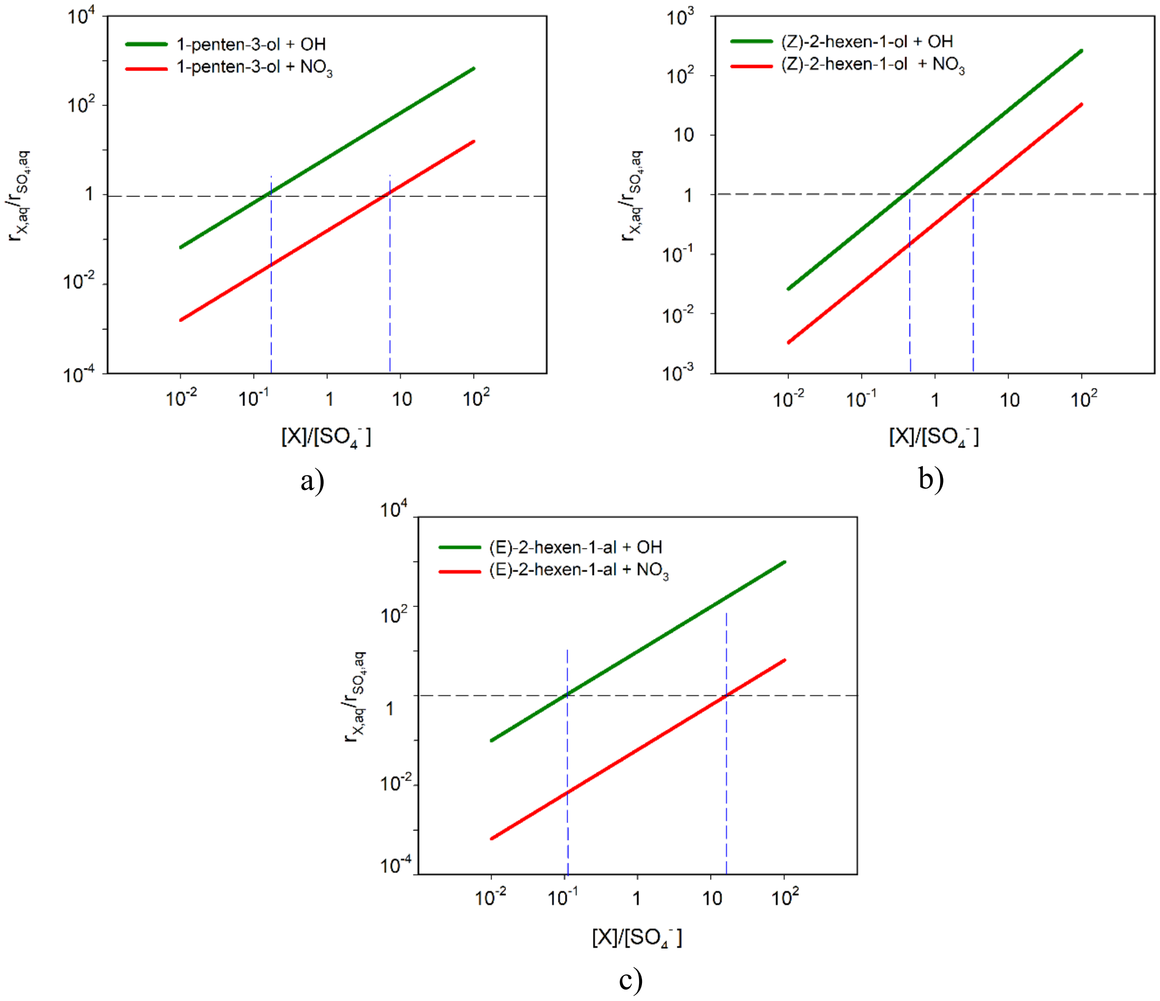

Figure S9. Influence of the radical ratio on the relative rates of GLVs removal from the atmosphere by the aqueous-phase reaction with $^{\bullet}OH$ or $NO_3^{\bullet}$ radicals and by the aqueous-phase reactions with $SO_4^{\bullet -}$ radical-anions: (a) PENTOL, (b) HEXOL, (c) HEXAL (Equation S21).

Figure S9 shows the ratios calculated with equation (S21) for various ratios of the radicals. Aqueous-phase reaction of PENTOL with $SO_4^{\bullet -}$ dominates over the aqueous-phase reaction with $^{\bullet}OH$ radicals provided $[^{\bullet}OH]/[SO_4^{\bullet -}] < 0.15$, and dominates over the aqueous-phase reaction with

$NO_3^{\bullet}$ provided $[NO_3^{\bullet}]/[SO_4^{\bullet-}] < 9$. Similarly, the conditions at which the reaction of $SO_4^{\bullet-}$ radical-anions with HEXOL dominate are: $[^{\bullet}OH]/[SO_4^{\bullet-}] < 0.8$ and $[NO_3^{\bullet}]/[SO_4^{\bullet-}] < 6$. For HEXAL the condition is: $[^{\bullet}OH]/[SO_4^{\bullet-}] < 0.11$ and $[NO_3^{\bullet}]/[SO_4^{\bullet-}] < 1.6$.

GLV partitioning between gas and aqueous phases. If the gas-aqueous partitioning of a GLV is governed by the Henry's Law equilibrium, the partitioning of that GLV between gas and aqueous phases is given by equation S22 and Fig. S10:

$$\frac{[GLV]_g}{[GLV']_{aq}} = \frac{[GLV]_g}{[GLV]_{aq}\omega} = \frac{1}{H_d\omega} \quad \text{(S22)}$$

where: [GLV']aq is the GLV concentration in the aqueous phase per volume of the gas phase, while other quantities were defined in previous sub-sections.

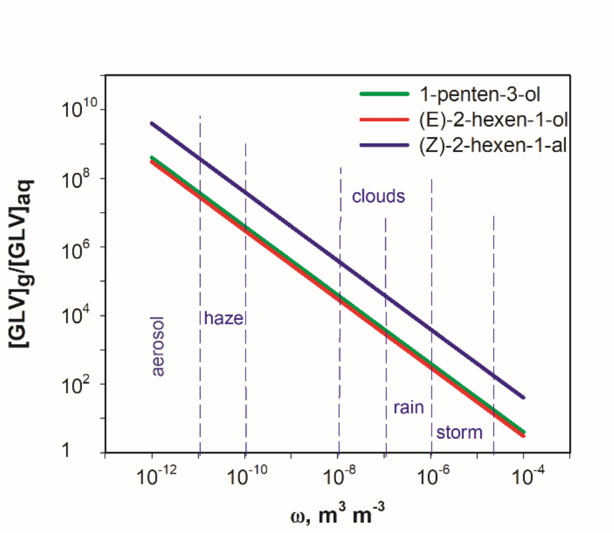

Figure S10. Gas-aqueous partitioning of GLV in various atmospheric systems.

Similar evaluation shows that the OH and NO_3 radicals reside predominantly in the gas phase while the $SO_4^{\bullet-}$ radicals – exclusively in the aqueous phase.

▪ 8. BIAS OF THE EXPERIMENTAL RATE CONSTANTS FOR $NO_3^{\bullet}$ REACTIONS WITH GLV.

Model_1

Model_1 was built to evaluate the bias of experimentally determined rate constants for reactions of GLVs with $NO_3^{\bullet}$ radicals. The bias resulted from the additional consumption of $NO_3^{\bullet}$ radicals in several reactions neglected in the experimental method (it assumed the radicals were consumed by a GLV only). The possible "competing" reactants included ^{-}OH, H_2O, $HO_2^{\bullet}$, and $S_2O_8^{2-}$ and

peroxy radicals derived from alkoxy radicals produced in reaction of $SO_4^{\bullet-}$ radicals with a GLV. Table S13 shows all reactions included in Model_1 along with rate constants and Arrhenius parameters. Most of the kinetic parameters were taken from the literature. The rate constants for reactions (13), (15), (24), and (25) in Table S13 were estimated in this work using the COmplex PAthway SImulator (COPASI) software (see the section below Table S13). The temperature-dependent rate constants used within the COPASI model for reaction 32 (Table S13) were determined experimentally within this study.

Table S13. Model_1 – reactions with temperature-dependent rate constants.

No	Reaction	*k* (298 K)	References	*A*	E_A	Ref., E_A (calc./estd.)
				L mol^{-1} s^{-1}	kJ mol^{-1}	
**1**	$H_2O \rightleftharpoons H^+ + OH^-$	→ 0.0014 s^{-1}<br>← 1.4×10^{11} L mol^{-1}s^{-1}	18	→ 9.9×10^{9}<br>← 5.5×10^{13}	73<br>15	19
**2**	$H_2SO_4 \rightleftharpoons H^+ + HSO_4^-$	→ 5.0×10^{13} s^{-1}<br>← 5.0×10^{10} L mol^{-1}s^{-1}	20	-	-	-
**3**	$HSO_4^- \rightleftharpoons H^+ + SO_4^{2-}$	→ 1.0×10^{9} s^{-1}<br>← 1.0×10^{11} L mol^{-1}s^{-1}	20	-	-	-
**4**	$HNO_3 \rightleftharpoons H^+ + NO_3^-$	→ 1.1×10^{12} s^{-1}<br>← 5.0×10^{10} L mol^{-1}s^{-1}	20	-	-	-
**5**	$HO_2 \rightleftharpoons H^+ + O_2^-$	→ 1.4×10^{6} s^{-1}<br>← 5.0×10^{10} L mol^{-1}s^{-1}	18	→ 1.3×10^{9}<br>← 5.1×10^{12}	17<br>12	19
**6**	$^{\bullet}OH + {}^{\bullet}OH \longrightarrow H_2O_2$	3.6×10^{9} L mol^{-1}s^{-1}	21	7.9×10^{10}	8	19
**7**	$^{\bullet}OH + H_2O_2 \longrightarrow H_2O + HO_2^{\bullet}$	2.7×10^{7} L mol^{-1}s^{-1}	293 K, 22	5.3×10^{8}	7	22
**8**	$^{\bullet}OH + HO_2^{\bullet} \longrightarrow H_2O + O_2^{\bullet-}$	6.0×10^{9} L mol^{-1}s^{-1}	21	8.2×10^{11}	12	21
**9**	$^{\bullet}OH + O_2^{\bullet-} \longrightarrow OH^- + O_2$	1.1×10^{10} L mol^{-1}s^{-1}	22	2.9×10^{10}	2	19
**10**	$^{\bullet}OH + HSO_4^- \longrightarrow SO_4^{\bullet-} + H_2O$	3.5×10^{5} L mol^{-1}s^{-1}	23	2.0×10^{7}	10	c
**11**	$SO_4^{\bullet-} + H_2O \longrightarrow {}^{\bullet}OH + HSO_4^-$	7.9 L mol^{-1}s^{-1}	23	4.1×10^{3}	16	24
**12**	$SO_4^{\bullet-} + HNO_3 \longrightarrow NO_3^{\bullet} + HSO_4^-$	5.5×10^{5} L mol^{-1}s^{-1}	25	1.0×10^{8}	13	26
**13**	$SO_4^{\bullet-} + NO_3^- \longrightarrow NO_3^{\bullet} + SO_4^{2-}$	9.0×10^{4} L mol^{-1}s^{-1}<br>9.2×10^{4} L mol^{-1}s^{-1}	27<br>*	 4.1×10^{8}	 21	 *Table S14
**14**	$SO_4^{\bullet-} + HO_2^{\bullet} \longrightarrow HSO_4^- + O_2$	3.5×10^{9} L mol^{-1}s^{-1}	28	5.2×10^{9}	1	26

**15**	$SO_4^{\bullet-} + S_2O_8^{2-} \longrightarrow S_2O_8^{\bullet-} + SO_4^{2-}$	1.0×10^4 L mol^{-1}s^{-1}<br>1.4×10^4 L mol^{-1}s^{-1}	24<br>*	2.2×10^6	-60	*Table S15
**16**	$SO_4^{\bullet-} + {}^{\bullet}OH \longrightarrow HSO_5^-$	1.0×10^{10} L mol^{-1}s^{-1}	29	1.5×10^{10}	1	26
**17**	$SO_4^{\bullet-} + SO_4^{\bullet-} \longrightarrow S_2O_8^{2-}$	2.0×10^8 L mol^{-1}s^{-1}	24	4.0×10^8	2	24
**18**	$SO_4^{\bullet-} + HSO_5^- \longrightarrow HSO_4^- + SO_5^{\bullet-}$	1.0×10^5 L mol^{-1}s^{-1}	30	1.9×10^7	13	26
**19**	$SO_4^{\bullet-} + OH^- \longrightarrow SO_4^{2-} + {}^{\bullet}OH$	1.4×10^7 L mol^{-1}s^{-1}	31	1.8×10^9	12	26
**20**	$NO_3^{\bullet} + NO_3^{\bullet} \longrightarrow N_2O_6$	3.0×10^7 L mol^{-1}s^{-1}	32	6.0×10^7	2	d
**21**	$NO_3^{\bullet} + OH^- \longrightarrow {}^{\bullet}OH + NO_3^-$	8.2×10^7 L mol^{-1}s^{-1}	27	1.0×10^{10}	12	d
**22**	$NO_3^{\bullet} + H_2O \longrightarrow {}^{\bullet}OH + HNO_3$	0.9 M^{-1}s^{-1}	33	4.7×10^2	16	d
**23**	$NO_3^{\bullet} + HO_2^{\bullet} \longrightarrow NO_3^- + H^+ + O_2$	3.0×10^9 L mol^{-1}s^{-1}	28	4.5×10^9	1	d
**24**	$NO_3^{\bullet} + S_2O_8^{2-} \longrightarrow NO_3^- + S_2O_8^{\bullet-}$	1.0×10^4 L mol^{-1}s^{-1}<br>1.0×10^4 L mol^{-1}s^{-1}	24<br>*	3.6×10^7	27	*Table S16
**25**	$NO_3^{\bullet} \longrightarrow$ products	47 ± 19 s^{-1}	*Table S17	-	-	-
**26a**	$SO_4^{\bullet-}$ + PENTOL⟶PENTOL alkyl	9.4×10^8 L mol^{-1}s^{-1}	*Table 1	7.9×10^9	5	*Table 7
**26b**	$SO_4^{\bullet-}$ + HEXOL⟶HEXOL alkyl	2.5×10^9 L mol^{-1}s^{-1}	*Table 1	1.1×10^{11}	10	*Table 7
**26c**	$SO_4^{\bullet-}$ + HEXAL⟶HEXAL alkyl	4.8×10^8 L mol^{-1}s^{-1}	*Table 1	2.9×10^9	5	*Table 7
**27**	GLV alkyl + O_2⟶GLV peroxy	2.0×10^9 L mol^{-1}s^{-1}	34	8.5×10^{11}	15	35
**28**	2 GLV peroxy⟶GLV p	1.6×10^8 L mol^{-1}s^{-1}	36	5.9×10^8	3	37
**29**	${}^{\bullet}OH$+ GLV peroxy⟶GLV p1	9.6×10^{10} L mol^{-1}s^{-1}	b, 38	1.4×10^{11}	1	26
**30**	$NO_3^{\bullet}$+ GLV peroxy⟶GLV p2	1.2×10^9 L mol^{-1}s^{-1}	b, 39	1.8×10^9	1	26
**31**	$SO_4^{\bullet-}$ + GLV peroxy⟶GLV p3	3.5×10^9 L mol^{-1}s^{-1}	a, 28	5.2×10^9	1	26
**32a**	$NO_3^{\bullet}$ + PENTOL⟶PENTOL alkyl2	1.5×10^8 L mol^{-1}s^{-1}	*Table 3	1.5×10^{11}	17	*Table 7
**32b**	$NO_3^{\bullet}$ + HEXOL⟶HEXOL alkyl2	8.4×10^8 L mol^{-1}s^{-1}	*Table 3	3.8×10^{10}	9	*Table 7
**32c**	$NO_3^{\bullet}$ + HEXAL⟶HEXAL alkyl2	3.0×10^7 L mol^{-1}s^{-1}	*Table 3	3.2×10^{10}	17	*Table 7

[a] adopted from the reaction of $SO_4^{\bullet-}$ with $HO_2^{\bullet}$; [b] adopted from gas-phase reactions of $RO_2^{\bullet}$ radicals, *This work; [c] an average of the activation energy of reaction 7 and 8; [d] Ea from corresponding reactions of $SO_4^{\bullet-}$ radicals, and A values calculated using the rate constants at 298 K

Kinetic parameters for reactions (13), (15), (24), and (25) in Table S13

The kinetic parameters for reactions

$$SO_4^{\cdot-} + NO_3^- \longrightarrow NO_3^{\bullet} + SO_4^{2-} \quad (13)$$

$$SO_4^{\cdot-} + S_2O_8^{2-} \longrightarrow S_2O_8^{\cdot-} + SO_4^{2-} \quad (15)$$

$$NO_3^{\bullet} + S_2O_8^{2-} \longrightarrow NO_3^- + S_2O_8^{\bullet-} \quad (24)$$

$$NO_3^{\bullet} \longrightarrow \text{products} \quad (25)$$

were estimated in this work from the experimental data collected during the background blank runs which were done in the same way as regular GLV + $NO_3^{\bullet}$ runs but without a GLV added. We analyzed the data with COPASI software using a built-in evolutionary algorithm (200 generations and population size 20) or a trial-and-error approach. The results are presented in Tables S14 – S17 along with the corresponding Arrhenius equations S23 – S25.

Table S14. The temperature-dependent rate constants for reaction (13)

$SO_4^{\cdot-} + NO_3^- \longrightarrow NO_3^{\bullet} + SO_4^{2-}$ (13)				
k (L mol^{-1} s^{-1})				
**278 K**	**288 K**	**298 K**	**308 K**	**318 K**
**4.5×10^4**	6.4×10^4	9.0×10^4	1.2×10^5	1.7×10^5
**6.7×10^4**	8.4×10^4	9.1×10^4	8.4×10^4	-
**5.4×10^4**	7.0×10^4	9.5×10^4	1.5×10^5	1.9×10^5
**Average**				
**$(5.5 \pm 1.1)\times10^4$**	$(7.3 \pm 1.0)\times10^4$	$(9.2 \pm 0.3)\times10^4$	$(1.2 \pm 0.3)\times10^5$	$(1.8 \pm 0.1)\times10^5$

$$k(T) = 4.1 \times 10^8 \exp\left(-\frac{2490 \pm 210}{T}\right) L\, mol^{-1}\, s^{-1} \quad (S23)$$

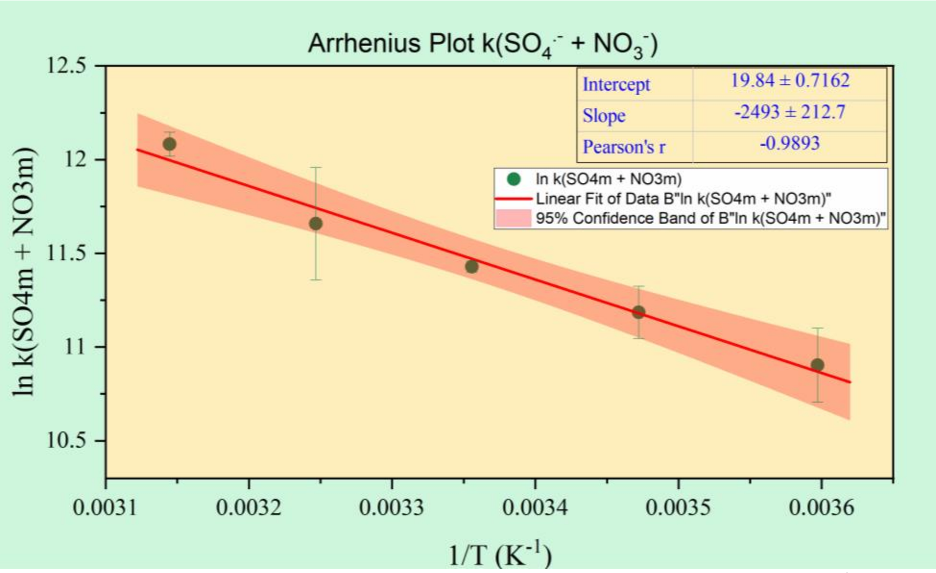

Figure S11: Arrhenius plot for reaction $SO_4^{\bullet-} + NO_3^- \longrightarrow NO_3^{\bullet} + SO_4^{2-}$ (13)

Table S15. The temperature-dependent rate constants for reaction (15).

$SO_4^{\bullet-} + S_2O_8^{2-} \longrightarrow S_2O_8^{\bullet-} + SO_4^{2-}$ (15)				
**k (L mol^{-1} s^{-1})**				
**278 K**	**288 K**	**298 K**	**308 K**	**318 K**
**5.0×10^4**	8.0×10^4	2.2×10^4	1.0×10^4	6.0×10^3
**5.0×10^4**	5.0×10^4	1.0×10^4	1.0×10^3	-
**7.6×10^4**	5.0×10^4	1.0×10^4	1.0×10^4	2.0×10^3
**Average**				
**$(5.9 \pm 1.5)\times10^4$**	$(6.0 \pm 1.7)\times10^4$	$(1.4 \pm 0.7)\times10^4$	$(7.0 \pm 5.2)\times10^3$	$(4.0 \pm 2.8)\times10^3$

$$k(T) = 2.2 \times 10^6 \exp\left(-\frac{-7190 \pm 1230}{T}\right) L\, mol^{-1}\, s^{-1} \quad \text{(S24)}$$

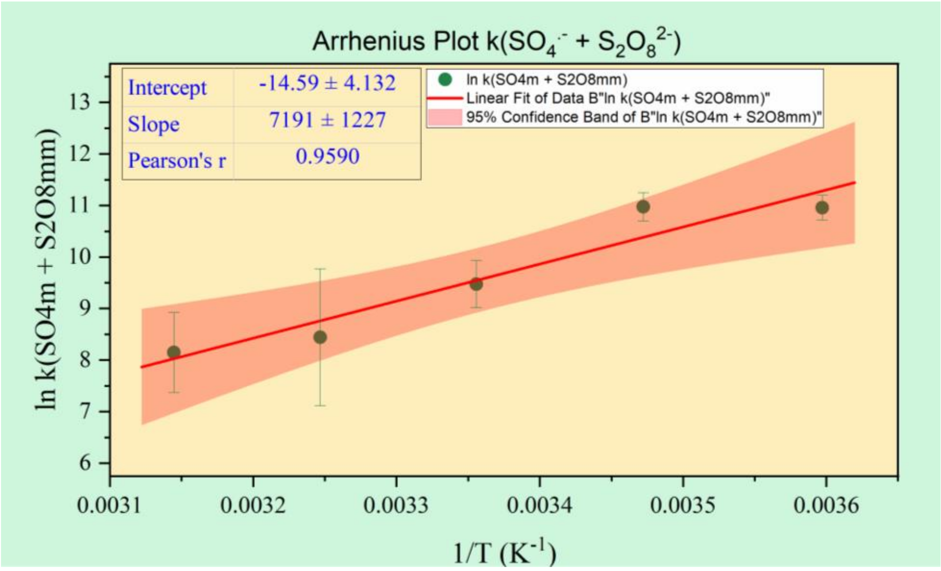

Figure S12: Arrhenius plot for reaction $SO_4^{\cdot-} + S_2O_8^{2-} \longrightarrow S_2O_8^{\cdot-} + SO_4^{2-}$ (15).

Table S16. The temperature-dependent rate constants for reaction (24).

$\mathbf{NO_3^{\bullet} + S_2O_8^{2-} \longrightarrow NO_3^- + S_2O_8^{\bullet-}}$ **(24)**				
**k (L mol^{-1} s^{-1})**				
**278 K**	**288 K**	**298 K**	**308 K**	**318 K**
**2×10^3**	3×10^3	1×10^4	1.6×10^4	4.0×10^4
**2×10^3**	4.4×10^3	1×10^4	1.8×10^3	-
**2×10^3**	6×10^3	1×10^4	1.6×10^4	3.0×10^4
**Average**				
**2×10^3**	(4.5 ± 2.5)×10^3	1.0×10^4	(1.7 ± 0.8)×10^4	(3.5 ± 2.1)×10^4

$$k(T) = 3.6 \times 10^7 \exp\left(-\frac{3200 \pm 900}{T}\right) L\ mol^{-1}\ s^{-1} \qquad \text{(S25)}$$

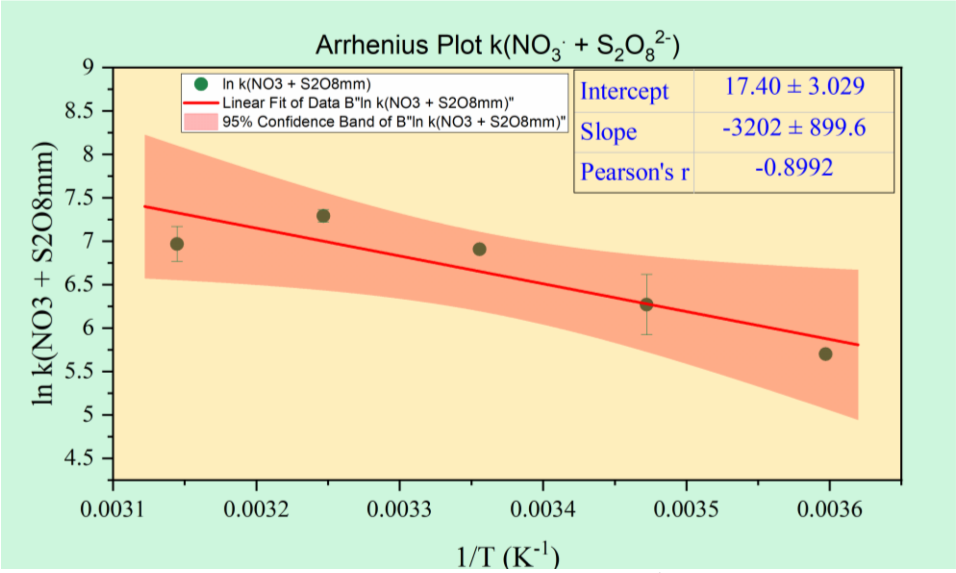

Figure S13: Arrhenius plot for reaction $NO_3^{\bullet} + S_2O_8^{2-} \longrightarrow NO_3^{-} + S_2O_8^{\bullet-}$ (24).

The first-order decay of $NO_3^{\bullet}$ radicals occurred in all experiments, but its rate did not depend on temperature. Probably, the sink can be explained through some physical processes like the wall loss of radicals. The magnitude of this sink was smaller than 10% of the total sink of $NO_3^{\bullet}$ without the reaction with GLV. Therefore, we suggest using the average value across the temperature range we studied.

Table S17. The estimation of the rate constants for the first-order sink of $NO_3^{\bullet}$ radicals.

**$NO_3^{\bullet} \longrightarrow$ products**					
**Average *k* (*s*$^{-1}$)**					
**278 K**	**288 K**	**298 K**	**308 K**	**318 K**	**278-318 K** average
**49**	67	32	64	25	47 ± 19

Estimation of the GLV + $NO_3^{\bullet}$ rate constants at T = 278 K, 288 K, 298 K. 308 K, and 318 K

The temperature-dependent second-order rate constants for the reaction of GLV with $NO_3^{\bullet}$ were estimated from the experimental data using Model_1 reactions and the COPASI parameter estimation task with the evolutionary programming method (number of generations 200, population size 20). The estimated values of rate constant k_{32} at individual temperatures are summarized in Tables S7-S9. The experimental and model traces are compared below in Figure S13-S25. According to the experimental conditions, the initial non-zero concentrations of reactants were: 0.03 mol L^{-1} for $S_2O_8^{2-}$ and 0.1 mol L^{-1} for NO_3^{-}. The initial $SO_4^{\bullet-}$ radical concentration was (2.3 ± 0.1) × 10^{-7} mol L^{-1} as calculated from the excimer laser's emitted energy in the experiments. The concentration of GLV was varied (0 – 1 × 10^{-4} mol $L^{-1}s^{-1}$) and is specified on the plots (Figure S13-S24).

A) 1-penten-3-ol + $NO_3^{\bullet}$

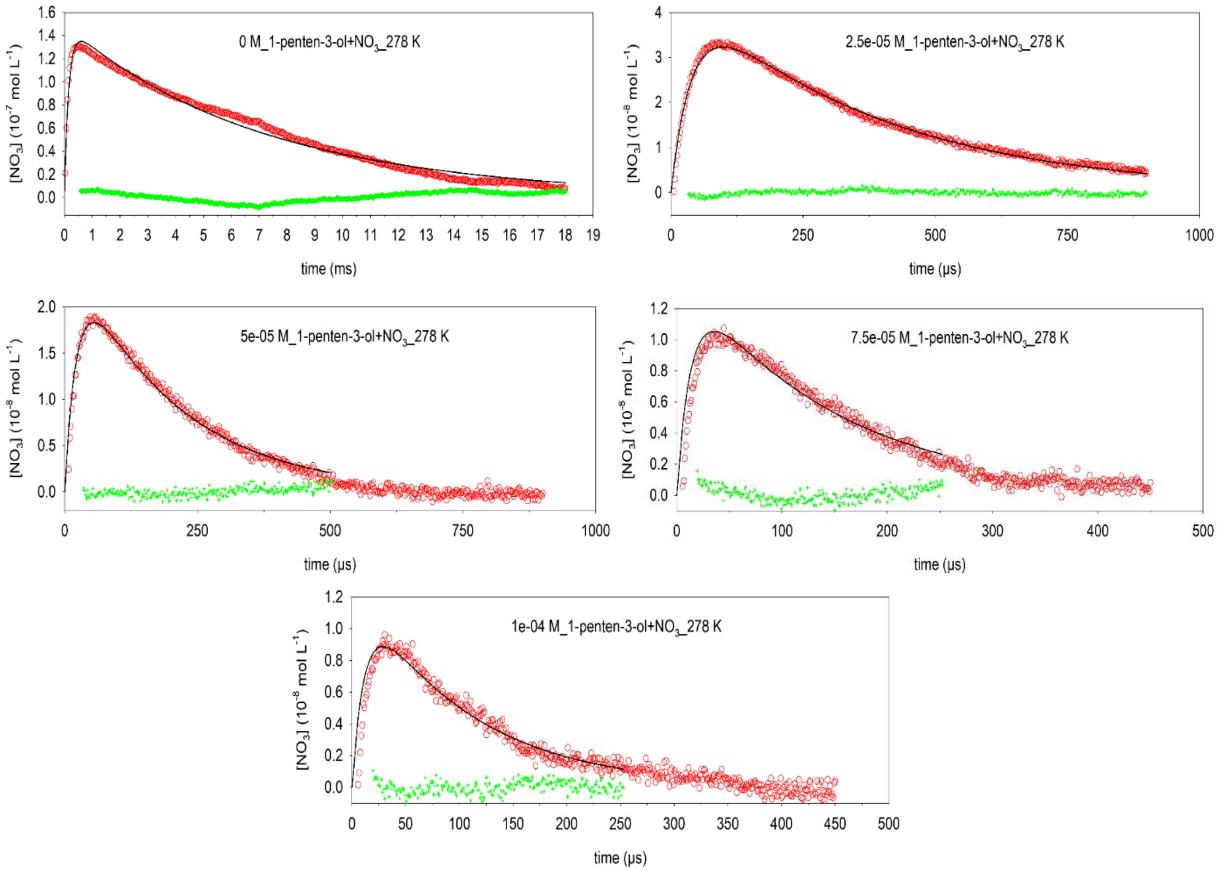

Figure S14. Concentration-time-profiles of $NO_3^{\bullet}$ in experiments at 278 K and various initial concentrations of 1-penten-3-ol (specified in the legends). Experimental data points are shown as red circles, simulation - as black lines, and residuals - as green crosses.

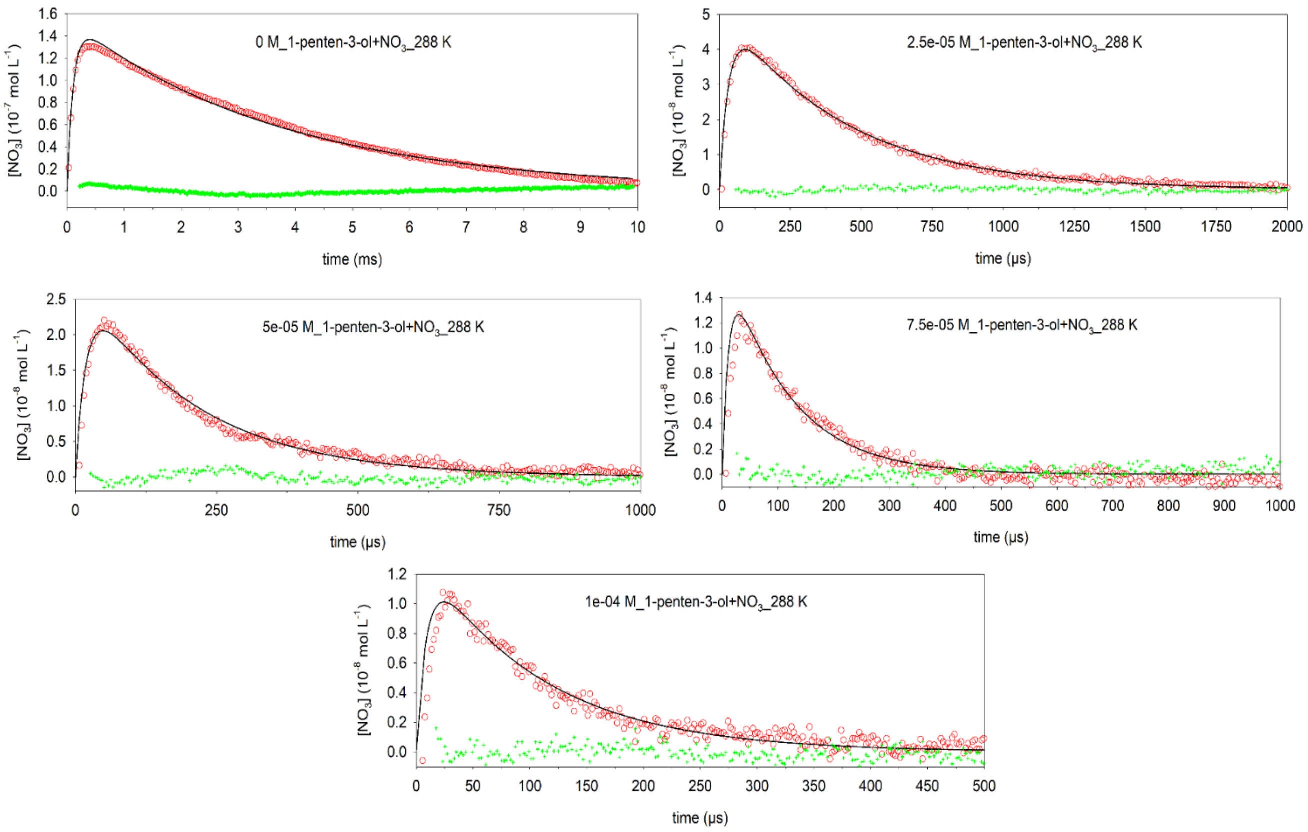

Figure S15. Concentration-time-profiles of $NO_3^{\bullet}$ in experiments at 288 K and various initial concentrations of 1-penten-3-ol (specified in the legends). Experimental data points are shown as red circles, simulation - as black lines, and residuals - as green crosses.

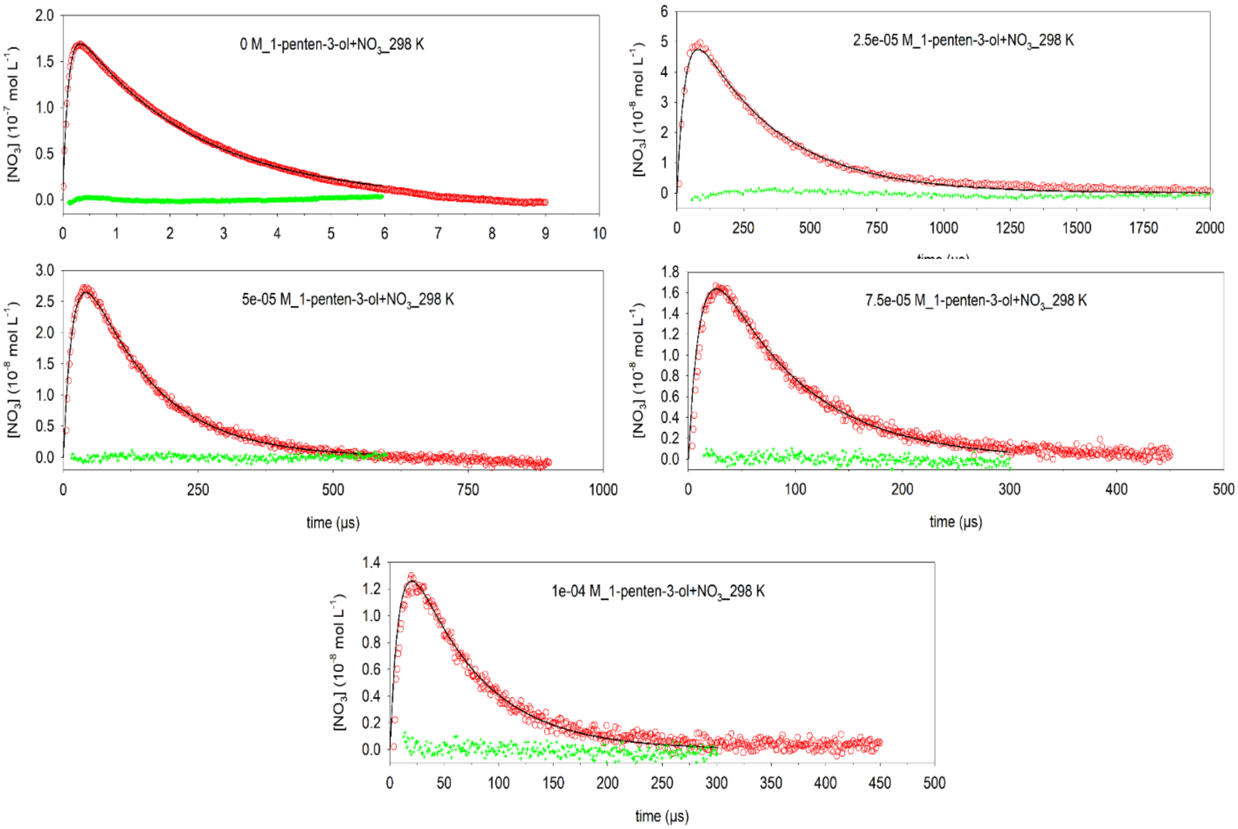

Figure S16. Concentration-time-profiles of $NO_3^{\bullet}$ in experiments at 298 K and various initial concentrations of 1-penten-3-ol (specified in the legends). Experimental data points are shown as red circles, simulation - as black lines, and residuals - as green crosses.

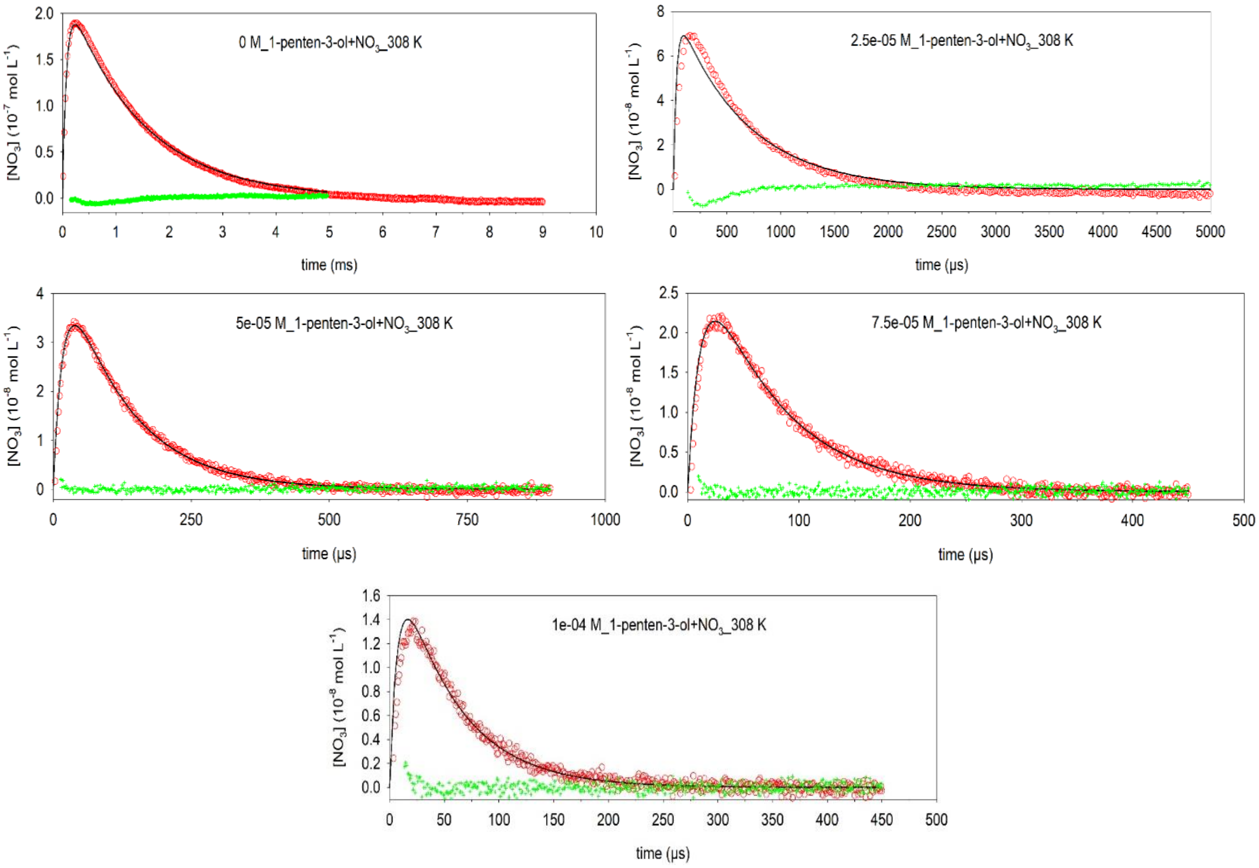

Figure S17. Concentration-time-profiles of $NO_3^{\bullet}$ in experiments at 308 K and various initial concentrations of 1-penten-3-ol (specified in the legends). Experimental data points are shown as red circles, simulation - as black lines, and residuals - as green crosses.

B) (Z)-2-hexen-1-ol + $NO_3^\bullet$

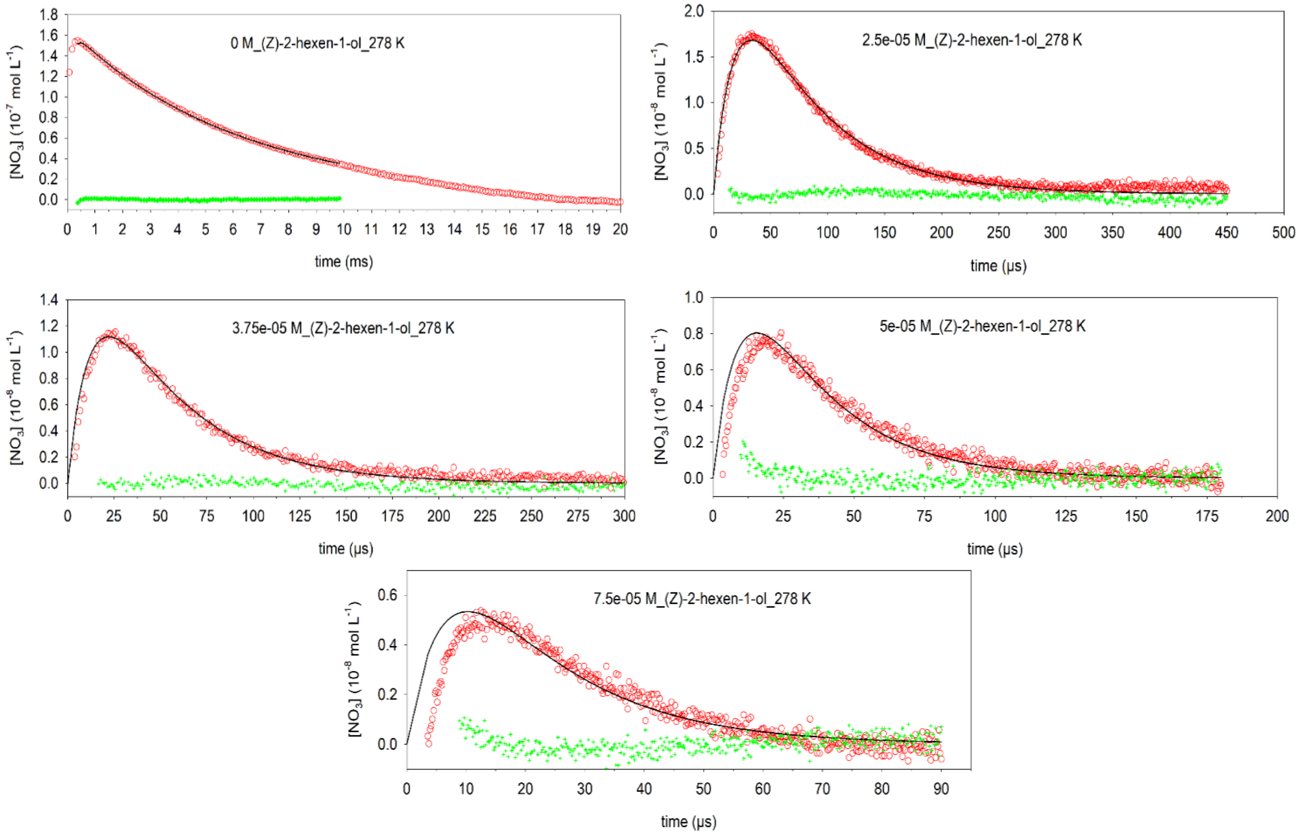

Figure S18. Concentration-time-profiles of $NO_3^\bullet$ in experiments at 278 K and various initial concentrations of (Z)-2-hexen-1-ol (specified in the legends). Experimental data points are shown as red circles, simulation - as black lines, and residuals - as green crosses.

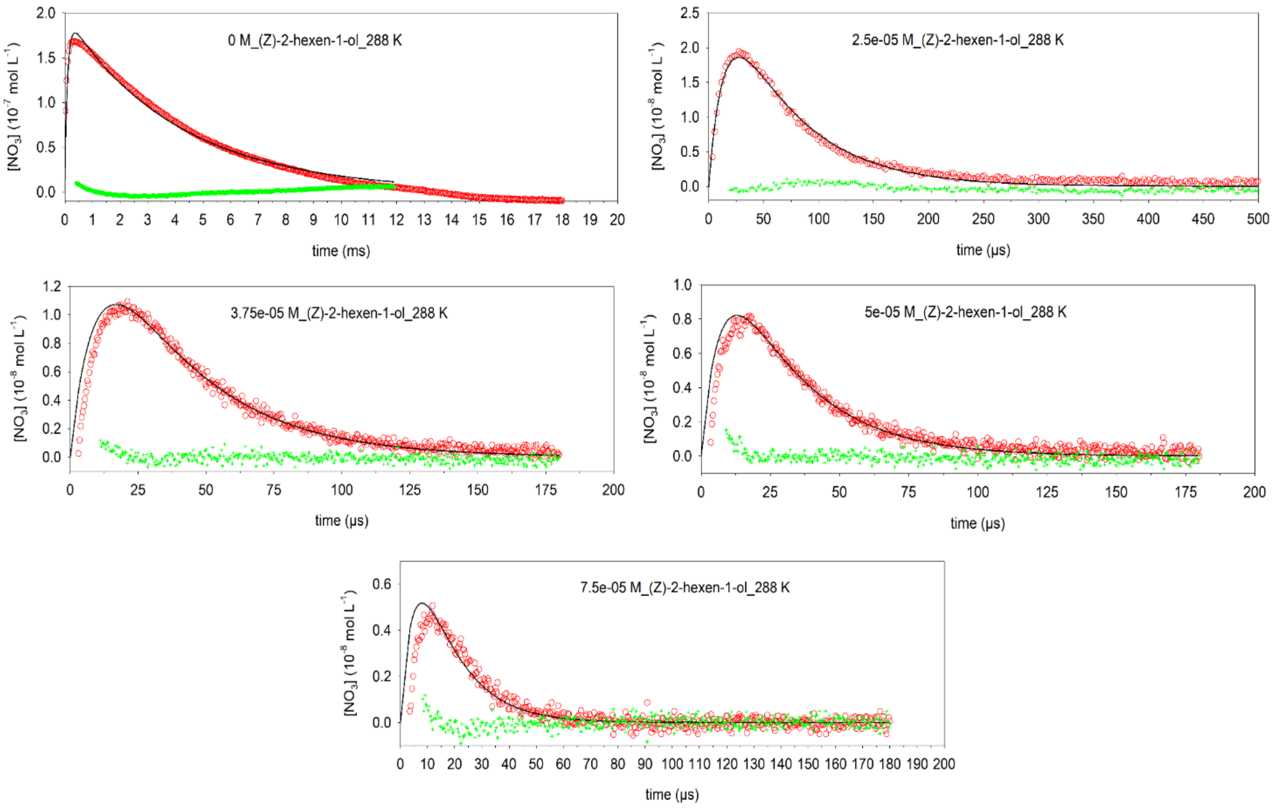

Figure S19. Concentration-time-profiles of $NO_3^{\bullet}$ in experiments at 288 K and various initial concentrations of (Z)-2-hexen-1-ol (specified in the legends). Experimental data points are shown as red circles, simulation - as black lines, and residuals - as green crosses.

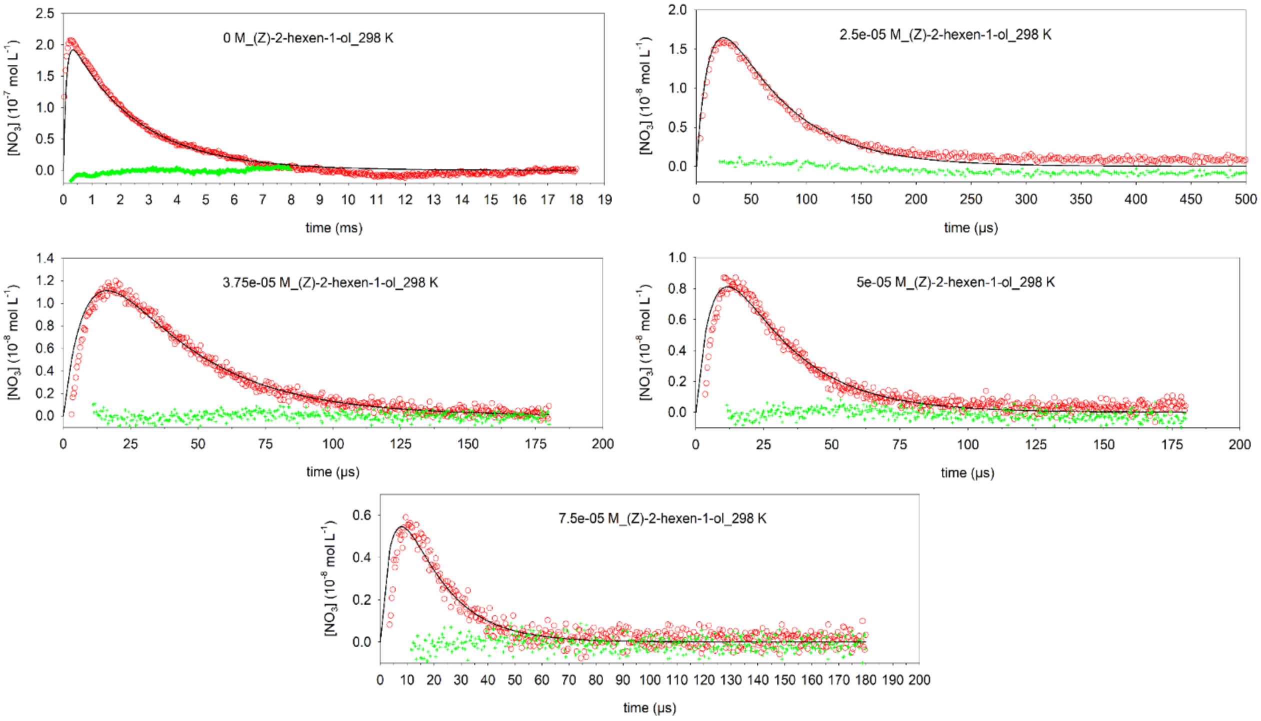

Figure S20. Concentration-time-profiles of $NO_3^{\bullet}$ in experiments at 298 K and various initial concentrations of (Z)-2-hexen-1-ol (specified in the legends). Experimental data points are shown as red circles, simulation - as black lines, and residuals - as green crosses.

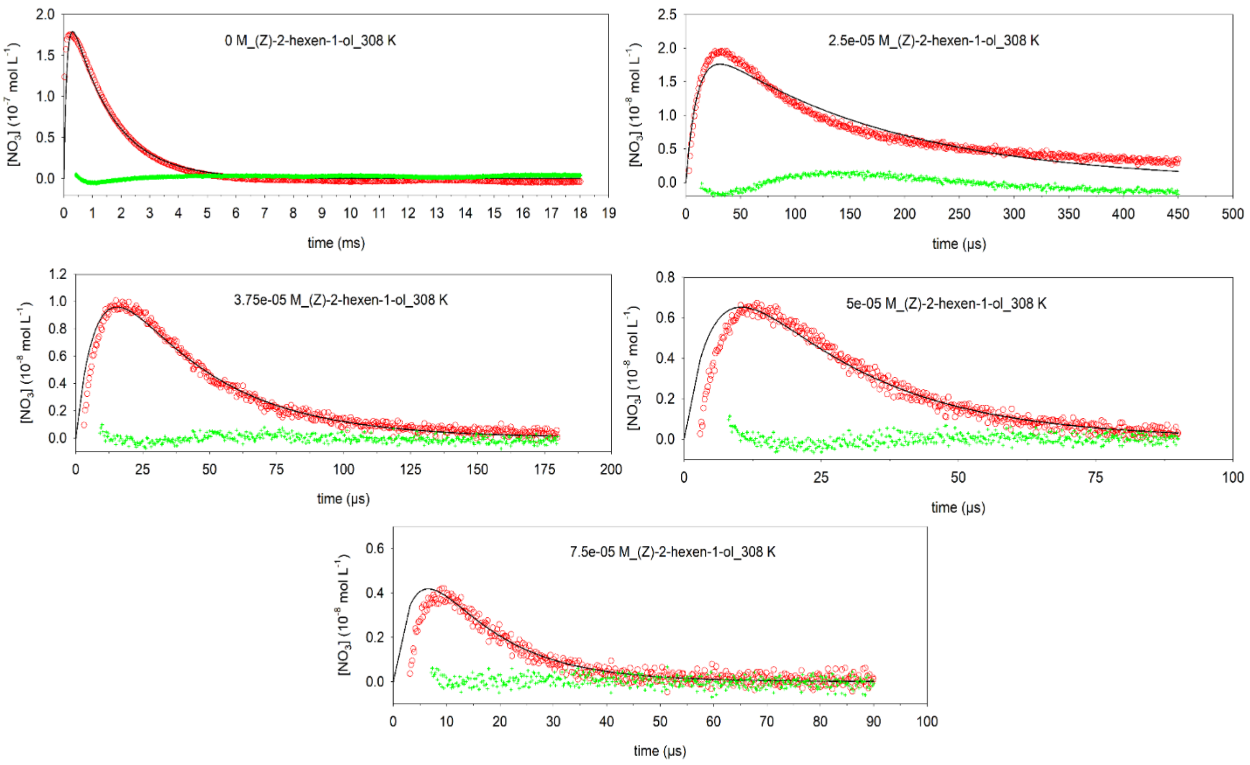

Figure S21. Concentration-time-profiles of $NO_3^{\bullet}$ in experiments at 308 K and various initial concentrations of (Z)-2-hexen-1-ol (specified in the legends). Experimental data points are shown as red circles, simulation - as black lines, and residuals - as green crosses.

C) (E)-2-hexen-1-al + $NO_3^{\bullet}$

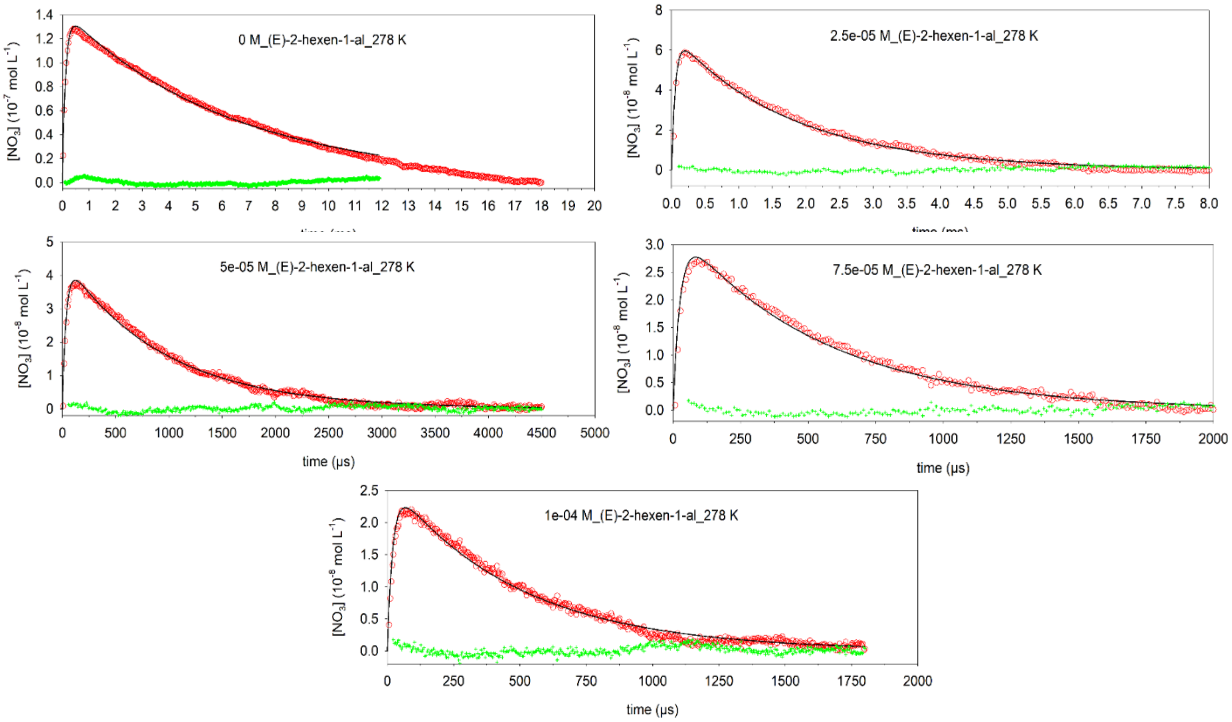

Figure S22. Concentration-time-profiles of $NO_3^{\bullet}$ in experiments at 278 K and various initial concentrations of (E)-2-hexen-1-al (specified in the legends). Experimental data points are shown as red circles, simulation - as black lines, and residuals - as green crosses.

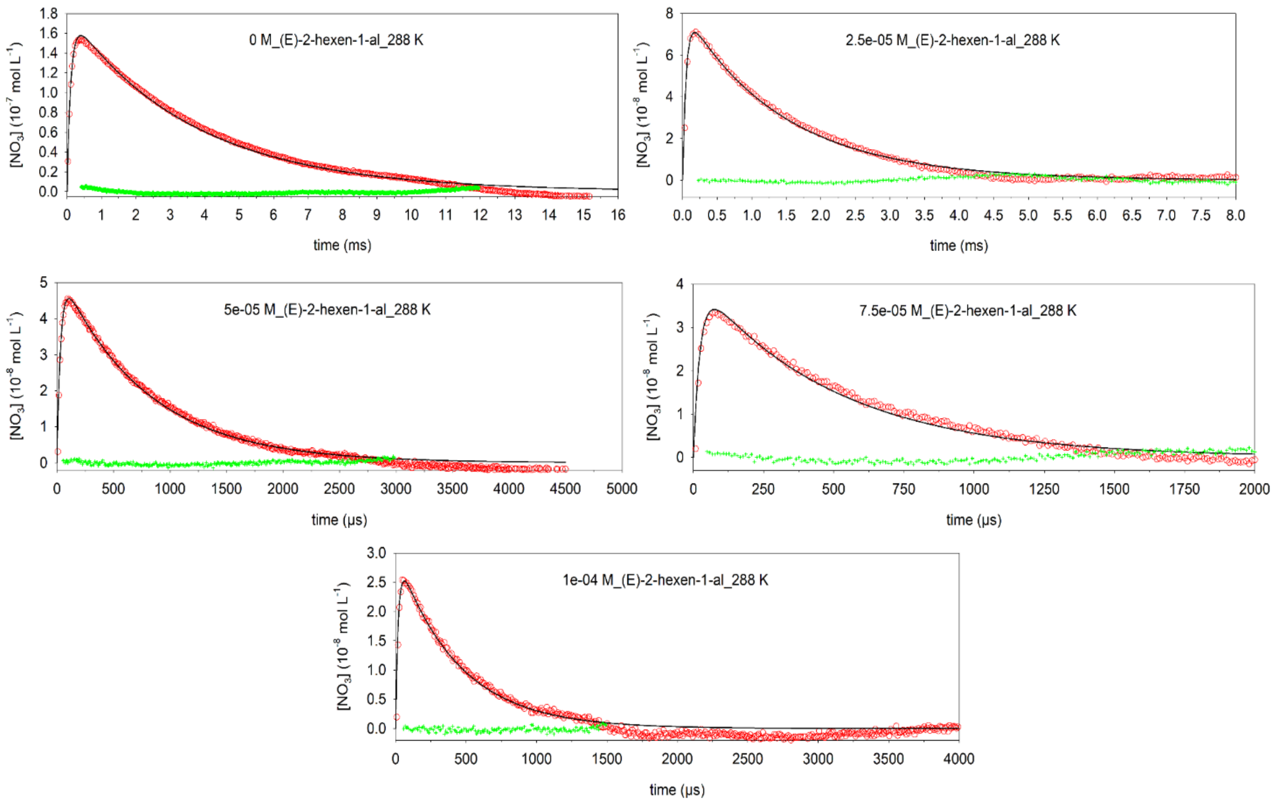

Figure S23. Concentration-time-profiles of $NO_3^{\bullet}$ in experiments at 288 K and various initial concentrations of (E)-2-hexen-1-al (specified in the legends). Experimental data points are shown as red circles, simulation - as black lines, and residuals - as green crosses.

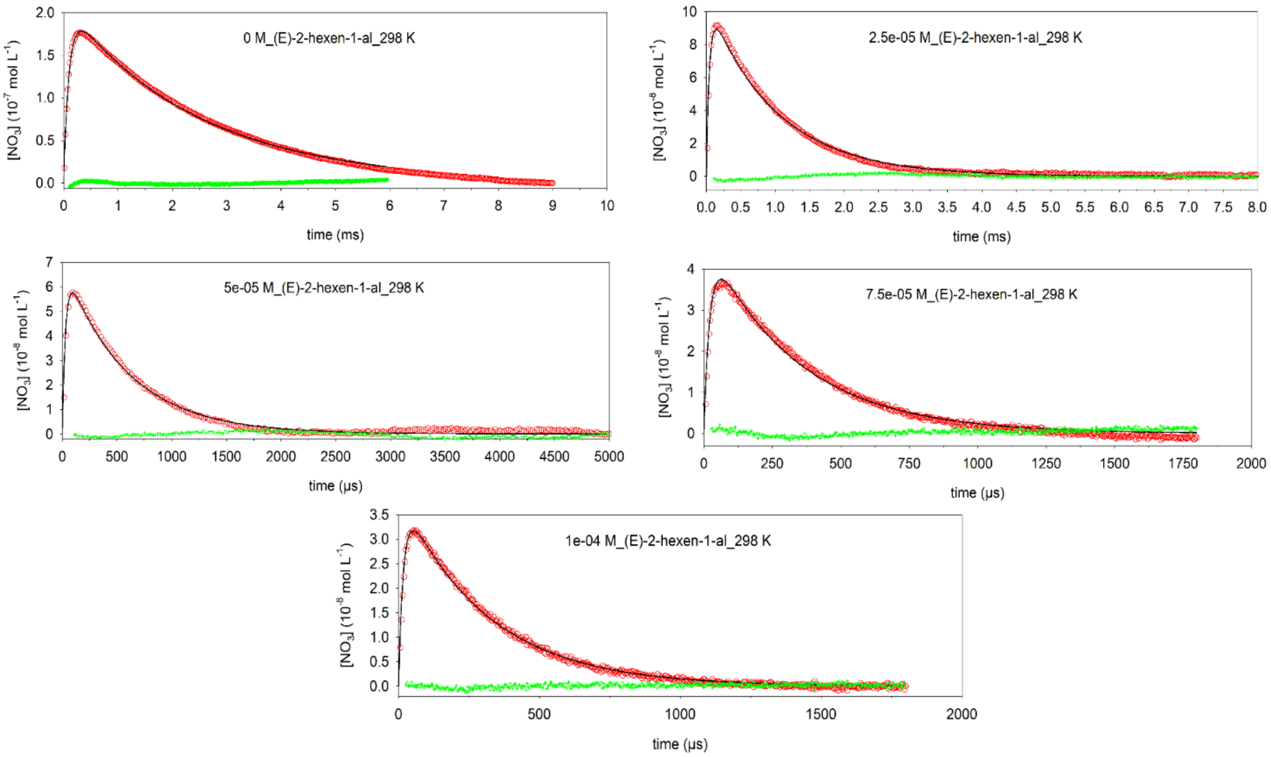

Figure S24. Concentration-time-profiles of $NO_3^{\bullet}$ in experiments at 298 K and various initial concentrations of (E)-2-hexen-1-al (specified in the legends). Experimental data points are shown as red circles, simulation - as black lines, and the residuals - as green crosses.

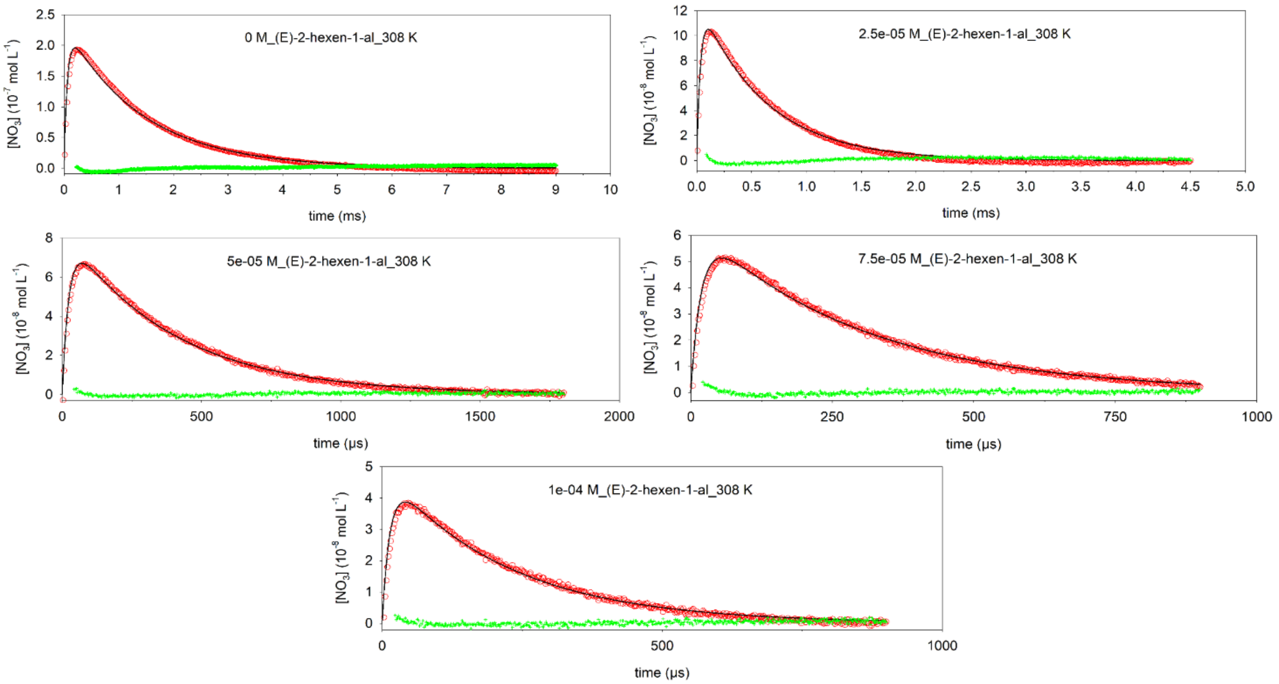

Figure S25. Concentration-time-profiles of $NO_3^{\bullet}$ in experiments at 308 K and various initial concentrations of (E)-2-hexen-1-al (specified in the legends). Experimental data points are shown as red circles, simulation - as black lines, and residuals - as green crosses.

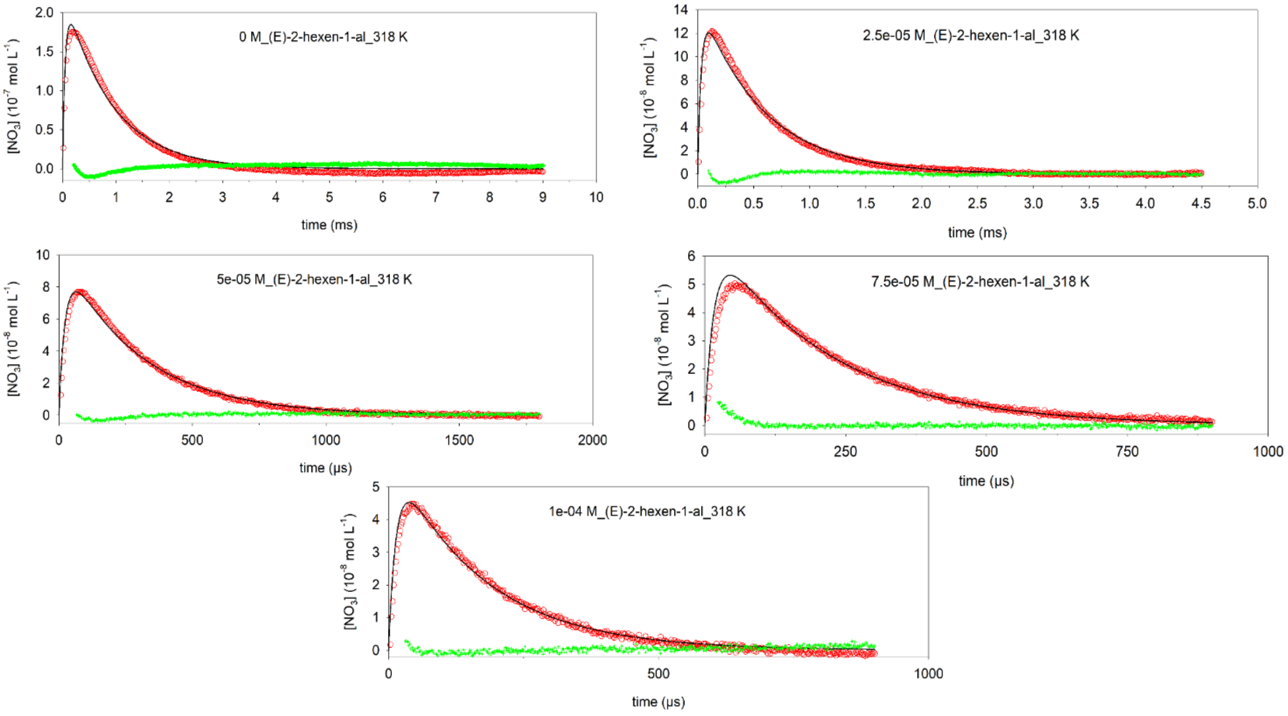

Figure S26. Concentration-time-profiles of $NO_3^{\bullet}$ in experiments at 318 K and various initial concentrations of (E)-2-hexen-1-al (specified in the legends). Experimental data points are shown as red circles, simulation - as black lines, and residuals - as green crosses.

- REFERENCES

1. US EPA. 2012, Estimation Programs Interface Suite™ for Microsoft® Windows, v 4.11. United States Environmental Protection Agency, Washington, DC, USA. In 2012.
2. Otto, T.; Schaefer, T.; Herrmann, H., Aqueous-Phase Oxidation of Terpene-Derived Acids by Atmospherically Relevant Radicals. *J. Phys. Chem. A* **2018,** *122*, (47), 9233-9241.
3. Schone, L.; Schindelka, J.; Szeremeta, E.; Schaefer, T.; Hoffmann, D.; Rudzinski, K. J.; Szmigielski, R.; Herrmann, H., Atmospheric aqueous phase radical chemistry of the isoprene oxidation products methacrolein, methyl vinyl ketone, methacrylic acid and acrylic acid - kinetics and product studies. *Phys. Chem. Chem. Phys.* **2014,** *16*, (13), 6257-6272.
4. He, L.; Schaefer, T.; Otto, T.; Kroflic, A.; Herrmann, H., Kinetic and Theoretical Study of the Atmospheric Aqueous-Phase Reactions of OH Radicals with Methoxyphenolic Compounds. *J. Phys. Chem. A* **2019,** *123*, (36), 7828-7838.
5. Smoluchowski, M. V., Versuch einer mathematischen Theorie der Koagulationskinetik kolloider Lösungen. *Z. Phys. Chem* **1918,** *XCII*, (92), 129-168.
6. Wilke, C. R.; Chang, P., Correlation of diffusion coefficients in dilute solutions. *Aiche J.* **1955,** *1*, (2), 264-270.
7. Joback, K. G.; Reid, R. C., Estimation of pure-component properties from group-contributions. *Chem. Eng. Commun.* **1987,** *57*, (1-6), 233-243.
8. Kojima, H.; Bard, A. J., Determination of rate constants for electroreduction of aromatic-compounds and their correlation with homogeneous electron-transfer rates. *J. Am. Chem. Soc.* **1975,** *97*, (22), 6317-6324.
9. Buxton, G. V.; Greenstock, C. L.; Helman, W. P.; Ross, A. B., Critical-review of rate constants for reactions of hydrated electrons, hydrogen-atoms and hydroxyl radicals ($^{\cdot}OH/^{\cdot}O^{-}$) in aqueous-solution *J. Phys. Chem. Ref. Data* **1988,** *17*, (2), 513-886.
10. Nightingale, E. R., Phenomenological theory of ion solvation-effective radii of hydrated ions. *J. Phys. Chem.* **1959,** *63*, (9), 1381-1387.
11. Bruce, E. P.; John, M. P.; John, P. O. C., *Properties of Gases and Liquids, Fifth Edition*. McGraw-Hill Education: New York, 2001.
12. Orlando, J. J.; Tyndall, G. S.; Ceazan, N., Rate coefficients and product yields from reaction of OH with 1-penten-3-ol, (Z)-2-penten-1-ol, and allyl alcohol (2-propen-1-ol). *J. Phys. Chem. A* **2001,** *105*, (14), 3564-3569.
13. Jimenez, E.; Lanza, B.; Antinolo, M.; Albaladejo, J., Photooxidation of Leaf-Wound Oxygenated Compounds, 1-Penten-3-ol, (Z)-3-Hexen-1-ol, and 1-Penten-3-one, Initiated by OH Radicals and Sunlight. *Environ. Sci. Technol.* **2009,** *43*, (6), 1831-1837.
14. Pfrang, C.; Romero, M. T. B.; Cabanas, B.; Canosa-Mas, C. E.; Villanueva, F.; Wayne, R. P., Night-time tropospheric chemistry of the unsaturated alcohols (Z)-pent-2-en-1-ol and pent-1-en-3-ol: Kinetic studies of reactions of NO_3 and N_2O_5 with stress-induced plant emissions. *Atmos. Environ.* **2007,** *41*, (8), 1652-1662.
15. Pfrang, C.; Martin, R. S.; Canosa-Mas, C. E.; Wayne, R. P., Gas-phase reactions of NO_3 and N_2O_5 with (Z)-hex-4-en-1-ol, (Z)-hex-3-en-1-ol ('leaf alcohol'), (E)-hex-3-en-1-ol, (Z)-hex-2-en-1-ol and (E)-hex-2-en-1-ol. *Phys. Chem. Chem. Phys.* **2006,** *8*, (3), 354-363.
16. Atkinson, R.; Arey, J.; Aschmann, S. M.; Corchnoy, S. B.; Shu, Y. H., Rate constants for the gas-phase reactions of cis-3-hexen-1-ol, cis-3-hexenylacetate, trans-2-hexenal, and linalool with OH and NO_3 radicals and O^{-3} at 296+/-2 K, and OH radical formation yields from the O^{-3} reactions. *Int. J. Chem. Kinet.* **1995,** *27*, (10), 941-955.
17. Herrmann, H.; Hoffmann, D.; Schaefer, T.; Brauer, P.; Tilgner, A., Tropospheric Aqueous-Phase Free-Radical Chemistry: Radical Sources, Spectra, Reaction Kinetics and Prediction Tools. *ChemPhysChem* **2010,** *11*, (18), 3796-3822.

18. Pastina, B.; LaVerne, J. A., Effect of molecular hydrogen on hydrogen peroxide in water radiolysis. *J. Phys. Chem. A* **2001,** *105*, (40), 9316-9322.
19. Elliot, A. J. *Rate constants and g-values for the simulation of the radiolysis of light water over the range 0-300 deg C*; Canada, 1994; p 69.
20. Graedel, T. E.; Weschler, C. J., Chemistry within aqueous atmsopheric aerosols and raindrops. *Rev. Geophys.* **1981,** *19*, (4), 505-539.
21. Elliot, A. J.; Buxton, G. V., Temperature-dependence of the reactions OH + O^{2-} AND OH + HO_2 in water upto 200-degrees-C. *J. Chem. Soc.-Faraday Trans.* **1992,** *88*, (17), 2465-2470.
22. Christensen, H.; Sehested, K.; Corfitzen, H., Reactions of hydroxyl radicals with hydrogen-peroxide at ambient and elevated-temperatures. *J. Phys. Chem.* **1982,** *86*, (9), 1588-1590.
23. Tang, Y.; Thorn, R. P.; Mauldin, R. L.; Wine, P. H., Kinetics and spectroscopy of the SO_4^- radical in aqueous-solution. *J. Photochem. Photobiol. A-Chem.* **1988,** *44*, (3), 243-258.
24. Bao, Z. C.; Barker, J. R., Temperature and ionic strength effects on some reactions involving sulfate radical SO4-(aq). *J. Phys. Chem.* **1996,** *100*, (23), 9780-9787.
25. Neta, P.; Huie, R. E., Rate constants for reaction of NO_3 radicals in aqueous-solutions. *J. Phys. Chem.* **1986,** *90*, (19), 4644-4648.
26. Herrmann, H. Photochemische Bildung, Spektroskopie und Kinetik freier Radikale in wässriger Lösung. University of Essen, 1998.
27. Exner, M.; Herrmann, H.; Zellner, R., Laser-based studies of reactions of the nitrate radical in aqueous-solution. *Ber. Bunsen-Ges. Phys. Chem. Chem. Phys.* **1992,** *96*, (3), 470-477.
28. Jiang, P. Y.; Katsumura, Y.; Nagaishi, R.; Domae, M.; Ishikawa, K.; Ishigure, K.; Yoshida, Y., Pulse-radiolysis study of concentrated sulfuric-acid-solutions-formation mechanism, yield and reactivity of sulfate radicals. *J. Chem. Soc.-Faraday Trans.* **1992,** *88*, (12), 1653-1658.
29. Klaning, U. K.; Sehested, K.; Appelman, E. H., Laser flash-photolysis and pulse-radiolysis of aqueous-solutions of the fluoroxysulfate ion, SO_4F. *Inorg. Chem.* **1991,** *30*, (18), 3582-3584.
30. Maruthamuthu, P.; Neta, P., Radiolytic chain decomposition of peroxomonophosphoric and peroxomonosulfuric acids. *J. Phys. Chem.* **1977,** *81*, (10), 937-940.
31. Herrmann, H.; Reese, A.; Zellner, R., Time-resolved UV/Vis diode-array absorption-spectroscopy of SO_X^-(X=3, 4, 5) radical-anions in aqueous-solution. *J. Mol. Struct.* **1995,** *348*, 183-186.
32. Wine, P. H.; Mauldin, R. L.; Thorn, R. P., Kinetics and spectroscopy of the NO3 radical in aqueous ceric nitrate nitric-acid solutions. *J. Phys. Chem.* **1988,** *92*, (5), 1156-1162.
33. Herrmann, H., Kinetics of aqueous phase reactions relevant for atmospheric chemistry. *Chem. Rev.* **2003,** *103*, (12), 4691-4716.
34. Neta, P.; Huie, R. E.; Ross, A. B., Rate constants for reactions of peroxyl radicals in fluid solutions. *J. Phys. Chem. Ref. Data* **1990,** *19*, (2), 413-513.
35. Schaefer, T.; Schindelka, J.; Hoffmann, D.; Herrmann, H., Laboratory Kinetic and Mechanistic Studies on the OH-Initiated Oxidation of Acetone in Aqueous Solution. *J. Phys. Chem. A* **2012,** *116*, (24), 6317-6326.
36. *Peroxyl radicals / edited by Zeev B. Alfassi*. Wiley: Chichester ;, 1997.
37. Tyndall, G. S.; Cox, R. A.; Granier, C.; Lesclaux, R.; Moortgat, G. K.; Pilling, M. J.; Ravishankara, A. R.; Wallington, T. J., Atmospheric chemistry of small organic peroxy radicals. *J. Geophys. Res.-Atmos.* **2001,** *106*, (D11), 12157-12182.
38. Assaf, E.; Song, B.; Tomas, A.; Schoemaecker, C.; Fittschen, C., Rate Constant of the Reaction between CH3O2 Radicals and OH Radicals Revisited. *J. Phys. Chem. A* **2016,** *120*, (45), 8923-8932.

39. Vaughan, S.; Canosa-Mas, C. E.; Pfrang, C.; Shallcross, D. E.; Watson, L.; Wayne, R. P., Kinetic studies of reactions of the nitrate radical (NO3) with peroxy radicals (RO2): an indirect source of OH at night? *Phys. Chem. Chem. Phys.* **2006,** *8*, (32), 3749-3760.
